# The effect of mindfulness on the inflammatory, psychological and biomechanical domains of adult patients with low back pain: A randomized controlled clinical trial

**DOI:** 10.1371/journal.pone.0276734

**Published:** 2022-11-09

**Authors:** Gustavo G. Diez, Eduardo Anitua, Nazareth Castellanos, Carmelo Vázquez, Purificación Galindo-Villardón, Mohammad H. Alkhraisat

**Affiliations:** 1 Nirakara Lab, Madrid, Spain; 2 Mindfulness and Cognitive Science Chair, Complutense University, Madrid, Spain; 3 University Institute of Regenerative Medicine and Oral Implantology - UIRMI (UPV/EHU-Fundacion Eduardo Anitua), Vitoria, Spain; 4 BTI Biotechnology Institute, Vitoria, Spain; 5 School of Psychology, Complutense University of Madrid, Madrid, Spain; 6 Faculty of Medicine, University of Salamanca, Salamanca, Spain; Goethe University Frankfurt: Goethe-Universitat Frankfurt am Main, GERMANY

## Abstract

**Objective:**

This study aims to study the effect of mindfulness-based program on the psychological, biomechanical and inflammatory domains of patients with chronic low back pain.

**Methods:**

A multicentre randomized and controlled clinical trial of parallel groups in patients with chronic low back pain between March 2019 to March 2020. Participants with no experience in mindfulness based intervention, were randomized to receive (36 patients) or not (34 patients) mindfulness-based stress reduction program for chronic back pain (MBSR-CBP). The program was performed in 9 sessions. Patients with chronic low back pain due to symptomatic discopathy (degenerative disc disease or herniated disc) were included. The principal outcome was changes in the blood level of cortisol and cytokines (tumor necrosis factor- α (TNF- α), interleukin-1β (IL-1β), interleukin-6 (IL-6) and interleukin-17 (IL-17)). Secondary outcomes (psychological factors, pain, and quality of life) were measured by validated questionnaires.

**Results:**

Of the 96 randomized patients, 70 who completed the study were included in the analysis (mean [range] age: 53 [33–73] years; 66% females). MBSR-CBP stopped the increase in cortisol, and reduced pro-inflammatory cytokine IL-1β (p = 0.05). It reduced depression (p = 0.046) and stress (p = 0.0438), perceived pain (p < 0.0001), and limitations related to health (p < 0.0001). It also increased the physical function (p = 0.002) and sleep quality (p = 0.05). Furthermore, it significantly increased life satisfaction (0.006), well-being (p = 0.001) and vitality (p < 0.0001). It also increased self-compassion (p < 0.0001) and significantly reduced the overidentification (p<0.0001) and catastrophization (p = 0.002).

**Conclusions:**

MBSR-CBP could be part of a multidisciplinary approach in the management of patients suffering from chronic low back pain.

## Introduction

Chronic low back pain, as indicated by the Global Burden of Disease Study 2019, represents the fourth overall cause of burden in the age range of 25 to 49 years, the seventh in the age range of 10–24 years, and the sixth in the age range of 50–74 years [1]. Back and neck pain present the world’s largest disease burden related to years lived with disability [2, 3]. The overall point prevalence mean in the general population is estimated at approximately 7% for cervical pain and 14% for low back pain [4, 5], with high recurrence rate in both conditions [1]. The condition is considered ‘chronic’ when it persists for more than 3 months or when it stays for a longer time than is necessary for the recovery of a tissue lesion [6].

Identified risk factors of pain chronicity are age, genetic predisposition, sex, previous experience and attitude toward pain [7, 8]. Nonspecific pain could account for the 90–95% of cases with back pain [9], where it has not been possible to clearly identify the structure, the pathology or the real origin of the symptoms.

Chronic pain states are often accompanied by affective, emotional and cognitive disorders (e.g., anxiety, depression, sleep disorders and cognitive deficits) [10]. The standard definition of chronic pain, defined as pain experienced on most days or every day in the previous 3 months, lacks specificity. There are individuals that evolve favorably after a few months and other that suffer chronic pain for several months, altering their way of life considerably. Furthermore, there are different degrees of severity. There are patients living their lives in a normal way, with limitations, and others who can spend days in bed.

To address this problem The US National Pain Strategy proposed the concept of high-impact chronic pain (HICP). The aim of the definition is to better identify people with significant levels of interference with life (i.e. daily activities, work, exercise, etc.) [11]. HICP incorporates both disability and pain duration. Based on the 2011 National Health Interview Survey, the prevalence of HICP can be calculated in US population [12]. IPCA affected 4.8% of the US adult population, or about 10.6 million people, compared to 13.60%, or about 29.9 million people with chronic pain without limitation (CPWL). It was estimated that 7.9 million people, 74% of the CPWL population, had chronic (high intensity) back pain, and 19 million people, 63.5% of the CPWL population, had chronic back pain (without limitations). Approximately 41.5% of the population with chronic back pain have HICP, significantly limiting their quality of life [12].

In the United States neck and low back pain are the second-leading condition that increased its spending between 1996 and 2013, with $64.4 billion over 18 years [13]. Therefore, there is a need to find cost-effective treatments for these conditions. Degenerative disc disease is affecting 266 million patients (3.63%) with low back pain every year [14]. It is incidence in Europe has been estimated in 5.7%. The subset of symptomatic disc degeneration has affected 403 million new patients every year worldwide. The treatments of symptomatic disc degeneration vary from noninvasive (physical therapy, pain medication) to invasive surgical intervention [15]. Surgical approaches will be associated with risks of the surgery and may derive in repeated surgeries due to the increase in the stress received by the other segments. There is a need for interventions that combine strategies based on decreasing symptoms of stress and depression, increasing coping with pain and increasing physical activity, as all of these variables are predictors of improvement.

Interestingly, the implementation of cognitive behavior-based program has produced a reduction of the Oswestry Disability Index (ODI) in nonsurgical and surgical patients [16]. Furthermore, mindfulness-based therapy has produced a positive effect on pain beliefs and psychological well-being of patients with disk herniation [17]. Preoperative mindfulness-based stress reduction (MBSR) may have benefits in pain control for those patients undergoing spine surgery [18].

MBSR programs could be a good alliance in the clinical managements of patients with painful disk degeneration. In 1982, Kabat-Zinn employed an outpatient program based on the practice of mindfulness meditation for chronic pain patients that achieved a significant reduction in pain [19]. This improvement has been related to the detached observation, a basic learning in meditation practices, which would help to uncouple the sensory aspect of pain experience from the affective alarm reaction [19]. More randomized clinical trials have reported a clinical benefit of the MBSR in patients with low back pain [20–22]. The MBSR has been shown to be cost saving when compared with usual care alone for chronic lower back pain [23]. That seminal study has significantly expanded the employment of mindfulness-based stress reduction (MBSR) programs in medicine. Participation in such programs has shown to be effective to improve anxiety, depression and pain [24–26]. Burns et al. have shown in patients with chronic pain that MBSR has produced significant differences to the “usual care alone” on average by session 6 [27]. The positive effect of the MBSR program on depression symptoms could be mediated by the reduction in rumination [25]. Also, neurobiological research has shown that mindfulness interventions significantly affect cortisol levels, age-related DNA telomere maintenance activity and neuronal activity [26, 28, 29].

Furthermore, there are indications that reductions in stress are mediated by lowering the levels of pro-inflammatory cytokines (TNF-α, IL-1β, IL-6 and IL-17) and influencing factors of cell transcription and gene expression [30–32].

To study the efficacy of the MBSR program on the inflammatory, psychological and biomechanical domains of patients with low back pain and symptomatic discopathy or disk herniation, randomized controlled clinical trials have been designed and implemented. The null hypothesis has been that MBSR had no significant influence on the inflammatory biomarkers and symptoms in patients with chronic low back pain. This could be related to the absence of effect on patients’ approach toward pain, disability, well-being and overall subjective health.

## Materials and methods

### Trial design

A multicenter randomized and controlled clinical study with parallel arms was designed and implemented. The clinical trial was conducted from March 2019 to March 2020. Three independent centers Eduardo Anitua Foundation (Vitoria, Spain), Complutense de Madrid University (Madrid, Spain) and Nirakara Lab (Madrid, Spain)) participated in the clinical trial. The trial protocol was approved by the ethics committee for research with medicaments of the Basque Country, Spain (FIBEA-01-EC/18/MIND)) On the 9th of January, 2019. The clinical trial was registered on clinicaltrials.gov (ClinicalTrials.gov Identifier: NCT03911375). Written informed consent was obtained.

### Patients

**Fig 1:**
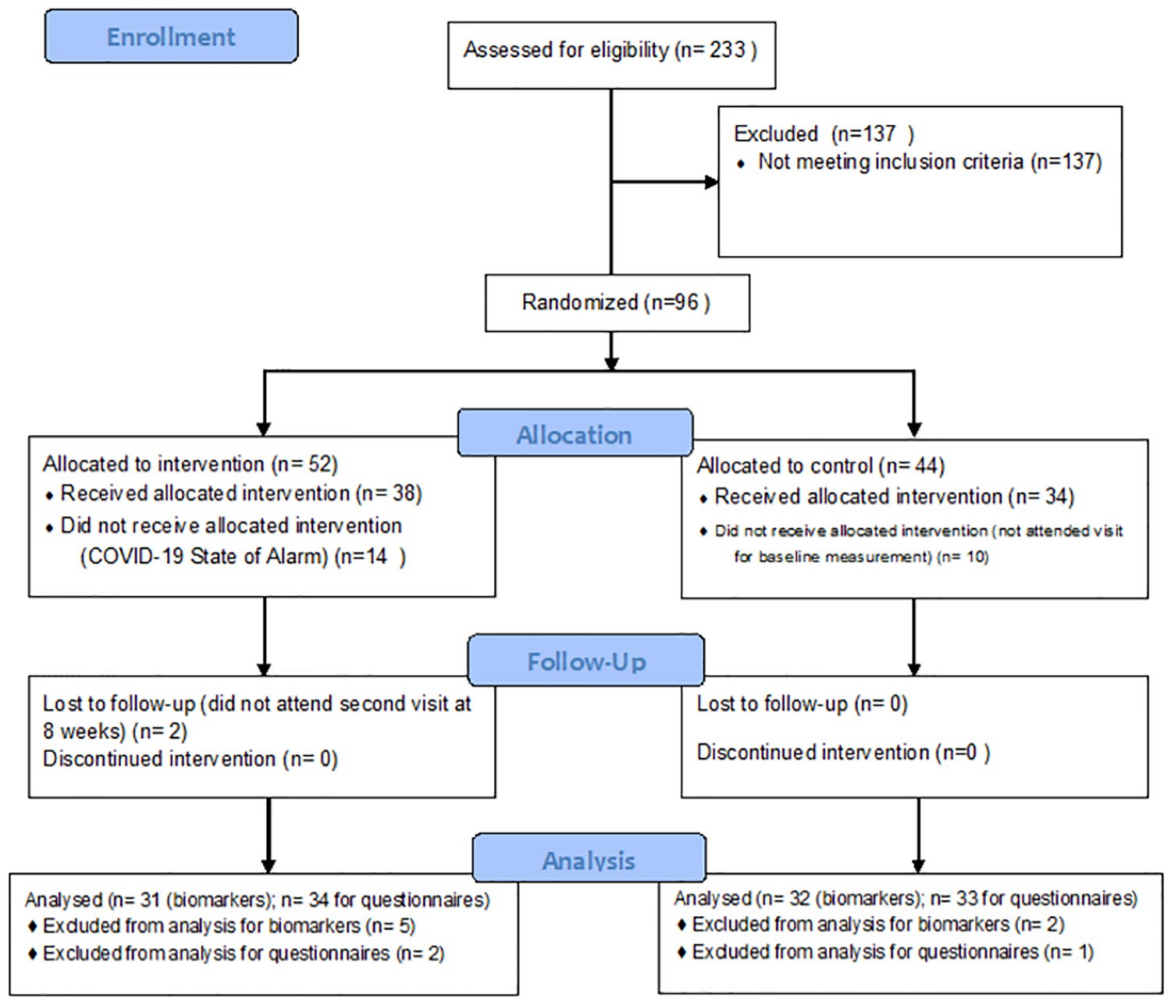
Study flow chart.

Invitations to participate in the free of cost study was sent via an email to the patients database of the participating Centers, describing the study objectives, the selection criteria and the commitment required from the patient. Those interested in participating (n = 233) filled out an online questionnaire, using the Qualtrics SAAS software featuring questions related to the exclusion criteria. The inclusion criteria were: having a diagnosis of symptomatic discopathy due to degenerative disc disease or herniated disc at any part of the spine, (i.e., participants had to provide a medical report informing of an existing discopathy), have been diagnosed with chronic pain no more than two years before the recruitment phase, normal or moderate mobility, normal cognitive state, have previously read and signed the informed consent form and ability and willingness to comply with the procedures included in the study. The exclusion criteria were serious psycho pathologies, suicidal thoughts, severe depression, psychosis, drug addiction, very limited functional capacity, and impaired cognitive functions (patients confined to their beds or a chair, or reliance on third-party), and initiate clinical, psychological or pharmacological treatments other than those used to treat pain or being experiencing drastic changes in lifestyle. Ninety six patients were initially recruited (Fig 1).

Fourteen patients could not make the program because of the restrictions installed in Spain by State of Alarm declaration. Another 10 patients did not attend the study claiming agenda problems. Two more patients did not attend the appointment for second evaluation. Seventy patients finally participated in the study with a total of 33 patients in Vitoria and 37 in Madrid. In the experimental group, 5 patients did not attend the visit for blood extraction and 2 patients did not deliver the questionnaires. For the control group, 2 patients did not attend the visit for blood extraction and 1 patient did not deliver the questionnaires. The sample had a mean age of 53 ± 10 years (range: 33 to 73 years) and 46 patients (66%) were women. The participants assigned to the control group continued with their ongoing pain treatment without any alteration.

### Randomization and allocation concealment

Patients randomization to the study group was performed by a specific computer program (Qualtrics software) and was performed independently for each of the centres [33]. Permuted block randomisation was done by GGD. Due to the characteristics of the experimental intervention, patients were not blinded. Only the statistical analyses were performed in a blinded way. Correspondence between the treatment and the patient number remained in a document kept by the principal investigator at each centre. The anonymized database for analysis only includes the patient number. This correspondence was communicated once data analysis was completed.

### Interventions

All participants (n = 70) continued with their own ongoing pain treatments. Data on the ongoing pain treatment was not collected. The experimental intervention was Mindfulness-based stress reduction program [34], but adapted to chronic back pain. It was a 30-hour program divided into 9 sessions at a frequency of 2.5 hours per week. An intensive session of 7.5 hours was performed between the sixth and seventh week. Pain coping skills training and sequential body movements were included. The sessions included theoretical and practical contents, in addition to detailed explanation of the exercises that patients needed to do at home. As homework, patients were instructed to follow a series of daily 45-min exercises which were specifically tailored for back pain conditions. The MBSR program was the one proposed by the Mindfulness Center of the University of Massachusetts [35], but the following modifications were included to adapt it to patients with chronic back pain. The two sessions related to body postures, originally proposed by Kabat-Zinn [34], were replaced by muscle relaxation and simpler movements supervised by two specialists in physiotherapy. Supplementary file (S1 File) describes in detail the exercises that had been implemented in this study. The purpose of this modification was to avoid unnecessary risks for patients by performing postures that could be a biomechanical challenge, to encourage mobility by starting from postures that would provide greater control and safety and to facilitate the development of mindfulness in the interoception of posture. Another modification in the MBSR program was the inclusion of psychoeducational contents on pain, the role of psychological distress in the development of pain and the use of effective coping strategies, with a focus on tolerance and acceptance. Thus, the adapted program paid more attention to the role of interoception and proprioception in the development of mindfulness. The instructor (GGD) in both sites had 10 years of experience teaching MBSR and was trained at the Center for Mindfulness at the University of Massachusetts Medical School. Patients were provided with an online platform where they had access to audio records for the daily exercises of MBSR, the workbook and the instructor to solve doubts related to the program. Questionnaires were administered every week to evaluate the adherence and possible adverse effects of the MBSR program. Patients’ attendance to the 9 sessions was also recorded.

### Outcomes

Demographic, psychological and biological variables were included in the study. Demographic and psychological variables were collected by means of an online questionnaire using Qualtrics software. Blood samples were taken at baseline and after 8 weeks. The samples were stored in a laboratory with offices in the two sites of the study and were analyzed on the same day to avoid bias. Furthermore, the samples were collected between 9:00–10:30 am. Cortisol was measured by chemiluminescent immunoassay (CLIA). The cytokines TNF-α, IL-1β, IL-6 y IL-17 were measured by multiplex immunoassay based on highly sensitive Luminex technique. All the biomechanical analysis was performed in an independent and accredited laboratory for clinical analysis.

Demographic variables included sex, education level and age. The Primary variables were stress reduction (measured by the variation of cortisol in the blood) and the variation of TNF-α, IL-1β, IL-6 y IL-17 levels in blood. The sleep and breathing analysis were also included and assessed; however, data are not included in this manuscript. Secondary outcomes were mindfulness, self-compassion, pain acceptance, disability, depression, anxiety, stress, well-being, health-related quality of life and life satisfaction. These variables were assessed by the following validated questionnaires:

Five Facets of Mindfulness Questionnaire—short form (FFMQ). This is a widely used measure of mindfulness that includes five factors: describing, acting with awareness, observing, not judging inner experience, and not reacting to inner experience. In addition, the total score is also used. The scale consists of 20 items rated on a 5-point Likert scale ranging from 1 to 5 [36].

Self‐Compassion Scale-Short Form (SCS). This is a measure of compassion for oneself. It measures the extent to which there is awareness of psychological distress and the degree of kindness towards oneself. It originally includes three components: mindfulness, self-compassion and common humanity [37]. The scale consists of 12 items rated on a 5-point Likert scale that is 5-point Likert scale ranging from 1 to 5.

Pain Catastrophizing Scale (PCS). Instrument designed to assess the coping strategies used by chronic pain patients [38]. The inventory includes 3 subscales that assess the following components: Rumination, Magnification and Helplessness. The scale consists of 13 items scored on a Likert scale from 1 to 5.

Chronic Pain Acceptance Questionnaire-Revised (CPAQ-R). It measures the acceptance of chronic pain in its psychosocial dimension [39]. Acceptance of pain focuses on the performance of meaningful activities and the achievement of important goals, rather than the use of internal resources to end pain unsuccessfully. It has two subscales: Activity engagement (pursuit of life activities regardless of pain) and Pain willingness (recognition that avoidance and control are often unworkable methods of adapting to chronic pain). Composed of 20 items, 7-point Likert scale.

Depression, Anxiety and Stress Scale—21 Items (DASS-21). A 21-item scale widely used to measure symptoms of depression, anxiety and stress [40]. The questions are asked with a one-week range. It uses a 4-point Likert scale with three subscales corresponding to symptoms of stress, anxiety and depression.

Satisfaction with Life questionnaire (SWLS). Life satisfaction is the personal perception of well-being or happiness; in other words, it is the valuation of one’s own life in relation to one’s own goals, expectations or interests directly mediated by the cultural context in which one lives [41]. The scale consists of five items and uses a Likert-type response format. Exploratory factor analysis suggests that the scale is unidimensional.

WHO‐5 Well Being Index. The World Health Organization’s WHO-5 index is a 5-item, 6-point Likert scale questionnaire that measures subjective well-being [42].

Short Form-36 Health Survey (SF-36). It is a generic scale that provides a profile of health status and is applicable to both patients and the general population [43]. The 36 items of the instrument cover the following scales: Physical Function, Physical Role, Bodily Pain, General Health, Vitality, Social Function, Emotional Role and Mental Health.

Pain and Sleep Questionnaire (PSQI). The 19 items of this test look at different determinants of sleep quality [44]. Items analyze different determinants of sleep quality, grouped into seven components: sleep quality, sleep latency, sleep duration, sleep efficiency, sleep disturbances, use of sleep medication and daytime dysfunction.

As a deviation from the original protocol, there were questionnaires (Pain Self-Efficacy Questionnaire (PSEQ), Survey of Pain Attitudes—Brief (SOPA.B), Pain Coping Inventory (PC), the Chronic Pain Grade Scale (CPGS;90) and Brief Pain Inventory (BPI)) were not passed to the patients to reduce the overloaded on them. Furthermore, the main coping strategy for chronic pain in MBSR-CBP is acceptance and detached attention to catastrophic thoughts (assessed with the PCS and CPAQ-R questionnaires). Having the vitality subscale provided by SF-36, the Brief Fatigue Inventory (BFI) was not passed. To measure stress, DASS was used and the Perceived Stressed Scale (PSS) was omitted.

### Statistical analysis

Sample size was calculated a priori using G power 3.1 [45]. Given a confidence level of 95%, a margin of error of 5% and assuming a normal distribution centered on an increase in stress reduction of 20–25% in the participants compared to the control group, a sample size of 96 participants is estimated. (48 per group with longitudinal measurements). To guarantee that the MBSR protocol is followed properly, a number of no more than 25 participants per course is advisable, subsequently, the experimental sample will be divided into two weekly groups of 24 subjects.

For the duration of the program, participants were asked not to change their clinical habits or initiate new pharmacological or clinical treatments. If they did, the participant continued to take part in the program, but he/she was not included in the analysis, to avoid interference with the variables being tested. Python 3.8.5 was used for preprocessing the questionnaire data and merging all the databases generated in the measurement procedure [33]. R version 4.0.1 (R Core Team, 2020) was used for the descriptive and comparative statistical analysis. A mixed 2 (Group) x 3 (Time) repeated-measures ANOVA was conducted to analyze changes in means over time. The assumption of normality was checked with Shapiro-Wilk test. The impact of outliers was checked for the analysis of each variable. Homogeneity of variance was checked by Levene’s test and the homogeneity of covariances by Box’s M test. The Greenhouse-Geisser sphericity correction was used for factors that violated the sphericity assumption. The mixed ANOVA was conducted using the rstatix package [46]. For those variables that did not pass the assumptions required for Mixed ANOVA, Robust ANOVA tests were used [47]. Specifically, for the normality analysis a double check was performed; Shapiro-Wilk normality test and visual check with QQ plot. Likewise, for variables that do not meet the assumptions, Dunn’s test for between-group comparisons and Wilcoxon test for within-group comparisons were used. For the analysis of correlations of cortisol with psychological variables, the Pingouin library in python and the skip function were used [48]. This function is a robust method that avoids possible errors produced by outliers [49]. The function yielded Spearman correlation coefficients after removing outliers. To explore the correlation between biological and psychological variables, the database was filtered by the experimental group, the difference of the variables after the intervention and before the intervention was calculated. This method captures the correlation of changes between variables. Statistical significance was set at p < 0.05.

## Results

Analyses of baseline differences between groups showed no significant differences in sex (*χ*^2^(1,*N* = 70) = 0.34, *p* = 0.56), age (*t*(64) = 16, *p* = .11), education level (*t*(66) = 0 .07, *p* = 0.94) and the RMQ disability score (*t*(61) = .49, *p* = .62) (Table 1). Supplementary file (S2 File) describes in detail the variables that had been measures in this study.

**Table 1:** The results of the assessment of blood biomarkers, sleep, disability, mindfulness and compassion, and psychological distress.

	Experimental group (Pre)			Experimental Group (post)			Control Group (Pre)			Control Group (Post)			Mixed ANOVA / Mixed Robust ANOVA	within group comparision(experimental)	within group comparision(control)
	count	mean	SD	count	mean	SD	count	mean	SD	count	mean	SD	p-value	p-value	p-value
Cortisol (μg/dL)	31	11.65	4.15	30	11.58	2.72	32	11.16	3.30	32	12.85	3.44	0.099	1.000	0.050
**immunological biomarkers**															
IL-1β (μg/L)	31	2.88	1.37	30	2.73	1.39	32	1.64	1.07	32	1.77	1.25	0.012	0.050	0.234
IL-6 (μg/L)	31	18.54	29.14	30	18.82	28.38	32	11.24	29.59	32	10.96	27.99	0.928	0.914	0.846
IL-17 (μg/L)	31	18.89	8.34	30	19.34	9.50	32	11.56	6.37	32	12.22	7.43	0.579	1.000	0.358
TNF-α (μg/L)	31	9.68	2.40	30	9.68	2.49	32	8.16	2.31	32	8.14	2.36	0.981	1.000	1.000
**Sleep**															
PSQI Subjective sleep quality	34	1.56	0.70	34	1.38	0.65	32	1.66	0.70	33	1.52	0.71	0.867	0.145	0.393
PSQI Sleep latency	34	1.47	1.08	34	1.29	0.94	32	1.94	0.91	33	1.94	0.90	0.570	0.162	0.685
PSQI Sleep duration	34	1.85	0.89	34	1.47	1.05	32	1.69	1.00	33	1.73	0.98	0.122	0.020	0.488
PSQI Sleep efficiency	34	1.26	1.08	34	1.15	1.21	32	1.28	1.25	33	1.15	1.15	0.974	0.309	0.684
PSQI Sleep disturbance	34	1.68	0.64	34	1.56	0.61	32	1.75	0.51	33	1.82	0.64	0.360	0.393	0.565
PSQI Use of sleep medication	34	0.88	1.34	34	1.00	1.37	32	1.38	1.31	33	1.58	1.32	0.834	0.952	0.174
PSQI Daytime dysfunction	34	1.35	0.65	34	0.94	0.81	32	1.41	0.87	33	1.30	0.98	0.251	0.005	0.536
PSQI Global	34	10.06	3.98	34	8.79	4.16	32	11.09	4.09	33	11.03	4.21	0.039	0.050	1.000
**Disability**															
RMQ	34	7.32	4.85	34	5.41	5.23	33	8.00	6.15	33	7.12	6.00	0.466	0.024	0.143
**Mindfulness and Compassion**															
FFMQ Aware actions	34	11.29	3.60	34	12.97	2.93	33	12.58	2.49	33	12.45	2.80	0.020	0.021	0.836
FFMQ Non-reactivity	34	11.26	2.53	34	12.53	2.96	33	11.82	1.89	33	11.79	2.00	0.015	0.004	1.000
FFMQ Non-judgmental inner experience	34	12.79	2.92	34	14.41	3.15	33	12.24	3.56	33	11.91	3.68	0.012	0.004	1.000
FFMQ Observation	34	12.21	2.25	34	14.47	2.56	33	14.18	2.24	33	14.06	3.06	0.001	0.001	1.000
FFMQ Description	34	12.24	1.42	34	12.26	1.60	33	12.00	2.00	33	11.39	1.90	0.191	1.000	0.210
FFMQ Global	34	59.79	6.56	34	66.65	8.33	33	62.82	7.54	33	61.61	7.43	0.000	0.000	0.554
SCS Self-Kindness	34	5.15	1.56	34	6.32	1.55	33	5.64	2.03	33	5.45	1.56	0.016	0.000	0.654
SCS Self-Judgment	34	5.85	1.64	34	7.12	1.93	33	5.24	2.21	33	5.18	1.72	0.023	0.006	1.000
SCS Common humanity	34	6.06	1.59	34	6.06	2.28	33	5.55	1.95	33	6.00	1.90	0.426	0.587	0.053
SCS Isolation	34	5.68	1.95	34	7.41	2.18	33	5.88	1.90	33	5.76	1.85	0.000	0.000	0.749
SCS Mindfulness	34	5.97	1.78	34	6.94	1.61	33	6.33	1.93	33	6.03	1.49	0.000	0.000	0.354
SCS Over-Identification	34	5.09	1.85	34	7.35	2.25	33	4.79	1.82	33	4.58	1.64	0.000	0.000	0.806
**Psychologycal Distress**															
DASS Depression	34	23.47	6.79	34	20.12	6.03	33	25.03	9.45	33	27.15	10.52	0.020	0.046	0.280
DASS Anxiety	34	21.47	6.10	34	19.59	3.96	33	23.52	7.55	33	25.45	7.99	0.063	0.268	0.386
DASS Stress	34	30.88	7.36	34	26.29	8.36	33	29.27	8.56	33	32.30	6.73	0.003	0.038	0.166
DASS Global	34	75.82	17.69	34	66.00	15.04	33	77.82	22.88	33	84.91	21.52	0.020	0.014	0.104

SD: Standard Deviation; IL: Interleukin; TNF: Tumor necrosis factor; PSQI: Pain and Sleep Questionnaire; RMQ: Roland-Morris Low Back Pain And Disability Questionnaire; FFMQ: Five Facets of Mindfulness Questionnaire; SCS: Self‐Compassion scale; DASS: Depression, Anxiety and Stress Scale.

### Stress reduction

Regarding the blood levels of cortisol, there were no significant differences between the study groups (F(1,59) = 2.81, p = 0.09). However, cortisol levels increased significantly in the control group only (F(1,31) = 5.577, p = 0.05) (Table 1). Robust correlation analysis shows that cortisol variations correlate significantly with SF-36 Pain (R(36) = -0.36, p = 0.037), with SF-36 Emotional well-being (R(36) = -0.42, p = 0.01), with SCS Mindfulness (R(36) = -0.56, p = 0.0005) and with SCS Self-Judgment (R(36) = -0.39, p = 0.027). Post-hoc analysis indicated that baseline values of IL-1, IL-17, and TNF-α were significantly higher in the experimental group (Table 1). These values remained higher (significant differences) in the experimental group after the mindfulness-based stress reduction program for chronic back pain (MBSR-CBP) program. Only in the experimental group, there was a statistically significant decrease in IL-1β (F(1,29) = 5.36, p = 0.050) before and after treatment. IL-6 did not show significant differences between the two study groups and over time.

Furthermore, the DASS-21 questionnaire was used to assess the negative mood in three subscales: stress, anxiety, and depression (Table 1). The baseline state of the negative mood state was similar between both groups. However, only patients in the experimental group showed a significant improvement. While the state of negative mood was decreasing in the experimental group, they were increasing in the control group (Table 1). Thus, the differences between the two groups were statistically significant. Similar results were also found in the subscales of stress (F(1,61) = 9.74, p = 0.003) and depression (F(1,61) = 5.74, p = 0.020). The anxiety subscale showed only significant differences between the two groups at the end of the study (F(1,65) = 14.63, p = 0.001).

### Mindfulness and self-compassion

Overall, patients in the control group did not experience changes in relation to the variables of mindfulness and self-compassion. However, patients in the experimental group showed statistically significant changes in relation to these two variables (Table 1).

Specifically, the level of mindfulness in the experimental group was lower (except for the subscales nonjudgment and description) than in the control group. The score of the "observation" subscale was significantly higher in the control group. ANOVA analysis showed a statistical significance interaction between the time and group factors in the subscales of nonreactivity (F(1,61) = 6.31, p = 0.015), nonjudgment (F(1,61) = 6.70, p = 0.012), observation (F(1,61) = 11.23, p = 0.001) and the global FFMQ score (F(1,61) = 24.60, p < 0.0001). Post-hoc analysis indicated that the MBSR-CBP program significantly improved mindfulness. Regarding the between-group comparison, differences were statistically significant after MBSR-CBP in the subscales of non-judgment and the global mindfulness score (Table 1). Regarding self-compassion, ANOVA analysis showed a statistical significance interaction between the time and group factors in the subscales of self-judgment (F(1,61) = 5.47, p = 0.023), isolation (F(1,61) = 15.71, p<0.0001), mindfulness (F(1,61) = 16.08, p = 0.0002) and overidentification (F(1,61) = 19.54, p<0.0001). Post-hoc analysis indicated that the MBSR-CBP program significantly improved in the values of these subscales (within-group comparison). Thus, the differences between the two study groups after MBSR-CBP practice were statistically significant.

### Sleep

Table 1 shows that the patients in the experimental group had better sleep quality as indicated by the global PSQI score.

### Patients approach toward pain

There is no significant interaction between groups and intervention in Pain Catastrophizing Scale (PCS) probably because both groups had significant changes before and after the program, as indicated by post-hoc analyses. Although the experimental group has significant changes in PCS, they cannot be attributed to the program (Table 2). The baseline comparison indicated the absence of statistically significant differences between groups. The overall CPAQ score increased in the experimental group while it decreased in the control group (Table 2). The differences were statistically significant in both groups at the end of the study, indicating an improvement in pain acceptance in patients in the experimental group (F(1,60) = 4.82, p = 0.032).

**Table 2:** The results of the assessment of general health, coping with pain and well-being.

	Experimental group (Pre)			Experimental Group (post)			Control Group (Pre)			Control Group (Post)			Mixed ANOVA / Mixed Robust ANOVA	within group comparision(experimental)	within group comparision(control)
	count	mean	SD	count	mean	SD	count	mean	SD	count	mean	SD	p-value	p-value	p-value
**General Healh**	**34**	**65.74**	**20.16**												
SF-36 Physical functioning	34	49.08	26.88	34	69.85	23.82	33	66.67	21.20	33	67.27	23.75	0.474	0.002	0.794
SF-36 Role limitations due to physical health	34	70.83	22.21	34	65.63	24.44	33	55.11	28.96	33	57.39	31.03	0.033	0.000	0.552
SF-36 Role limitations due to emotional problems	34	35.93	16.26	34	79.90	20.22	33	75.76	23.97	33	77.78	22.02	0.270	0.018	0.393
SF-36 Energy/fatigue	34	54.67	13.71	34	47.50	14.57	33	37.22	19.39	33	38.89	16.95	0.007	0.000	0.920
SF-36 Emotional well-being	34	58.46	25.32	34	62.02	14.93	33	50.08	17.32	33	50.04	15.40	0.137	0.062	1.000
SF-36 Social functioning	34	44.85	18.15	34	78.31	21.61	33	62.50	28.64	33	64.02	28.43	0.005	0.000	0.596
SF-36 Pain	34	47.06	15.91	34	56.40	22.34	33	43.26	25.77	33	43.94	26.21	0.007	0.000	1.000
SF-36 General health				34	51.91	15.42	33	45.45	23.33	33	46.67	21.42	0.106	0.134	1.000
**Coping with pain**	**34**	**28.88**	**6.08**												
PCS Rumination	34	7.29	2.56	34	9.44	4.02	33	10.97	4.07	33	10.15	4.24	0.415	0.204	0.342
PCS Magnification	34	14.03	5.06	34	5.85	2.38	33	7.09	2.52	33	6.36	2.40	0.333	0.002	0.102
PCS Helplessness	34	32.38	11.26	34	10.97	4.52	33	14.55	5.37	33	13.12	5.55	0.244	0.001	0.066
PCS Global	34	41.94	9.56	34	26.26	10.03	33	32.61	10.14	33	29.64	11.13	0.291	0.002	0.044
CPAQ Activity Engagement	34	39.29	6.16	34	47.32	8.81	32	42.22	12.16	33	42.24	12.01	0.047	0.014	1.000
CPAQ Willingness to Pain	34	81.24	10.27	34	38.09	6.45	32	40.16	8.49	33	36.64	7.71	0.219	0.702	0.038
CPAQ Global				34	85.41	10.19	32	82.38	14.94	33	78.88	12.70	0.032	0.114	0.440
**Well-being**	**34**	**19.97**	**5.54**												
SWLS	34	11.56	4.26	34	22.24	4.94	33	20.61	7.13	33	20.39	7.35	0.011	0.006	1.000
WHO-5				34	14.82	4.65	33	10.70	5.17	33	10.58	4.95	0.001	0.001	1.000

SD: Standard Deviation; SF-36: Short Form-36 Health Survey; PCS: Pain Catastrophizing Scale; CPAQ: Chronic Pain Acceptance Questionnaire; SWLS: Satisfaction with Life questionnaire; WHO: World Health Organization.

### Pain score

Pain assessment was carried out using the SF-36-pain scale, a 6-level Likert scale with 2 questions, the first assessing pain intensity and the second assessing the extent to which pain has interfered with daily activities. The results show significant differences in the combined time/group effect (F(1,61) = 7.70, p = 0.007), with a statistically significant decrease in pain in the experimental group (F(1,31) = 21.31, p = 0.0001) (Table 2).

### Disability

Although the intergroup comparison indicated no significant differences in the RMQ score, the within group analysis showed a significant reduction in the experimental group only (Table 1). Subscales of the SF-36 (Table 2), patients in the experimental group showed statistically significant improvements in the combined time/group effect in physical functioning (F(1,61) = 133.00, p = 0.024). However, the SF-36 subscale limitations due to physical health does show significant changes attributable to the interaction of both factors (F(1,61) = 4,77, p = 0.033).

### Well-being

The results show statistically significant changes in the combined time/group effect in WHO-5 (F(1,61) = 11.84, p = 0.001) and SWLS (F(1,61) = 6.95, p = 0.011). Furthermore, only in the experimental group did both WHO-5 (F(1, 31) = 15.79, p = 0.0008), and SWLS (F(1,31) = 10.64, p = 0.006) improved over time (Table 2).

### Overall subjective health

In addition to the SF-36 subscales described in previous sections, SF-36 showed significant changes in the combined time/group effect on other subscales of interest to the study sample (Table 2). These subscales were energy/fatigue (F(1,61) = (1,61 = 7.65, p = 0.007), and social functioning (F(1,61) = 8.36, p = 0.005). Post-hoc analysis showed that patients in the experimental group had improvement in these subscales over time: energy/fatigue (F(1,31) = 28.41, p <0.0001) and Social Functioning (F(1,61) = 287.00, p < 0.0001).

## Discussion

The effect of the MBSR program has not been statistically significant on the blood cortisol level. This could be attributed to the fact that all the patients in this study had the blood cortisol within the normal reference values (6–23 μg/dl) [50]. Nevertheless, MBSR has slowed down the increase in blood levels of cortisol (a measure of stress), considering its increase in patients in the control group. MBSR-CBP has significantly reduced blood levels of IL-1β. IL-1β is a proinflammatory and pronociceptive cytokine [51]. It has been reported to be involved in neurodegeneration, chronic inflammation, and chronic pain [52], but no evidence has been found for its association with chronic back pain [51]. However, the meta-analysis by Ng et al. showed that older people with depression had significantly higher peripheral IL-1β levels than people in the control group and along the same lines [53]. Ellul et al. concluded that IL-1β is a reliable biomarker for major depressive disorder [54]. The IL-1β level changes could be due to the effect of the program on depressive symptoms. Depression has been linked to a high accumulation of IL-1β [55–57]. In a study with a murine model, peripheral nerve injuries caused in mice increased the expression of proinflammatory cytokines, which in turn contributed to the development of depressive-like behaviors in the mice. Also, stress-induced in mice for two weeks promoted the occurrence of depressive-like behavior after nerve injury by promoting IL-1β expression [58]. Proinflammatory cytokines (IL-1β, IL-6 and TNF-α) may be screening biomarkers in the prediction of treatment response in patients with depression [56]. In addition, the relationship between back pain and depressive symptoms has also been reported by [59]. Overall, the Intervention seems to have a psychological effect by decreasing the symptoms of psychological distress and this could have been a cause of the decrease in IL-1β in the experimental group. Long-term follow-up is needed to find out the relationship between MBSR, cortisol, and inflammatory cytokines.

Direct measures related to mindfulness have significantly improved in patients in the experimental group thus suggesting that mindfulness training has had the expected effect. Patients reported an increase in mindfulness (FFMQ) and self-compassion (SF-SCS).

According to the review by Haldeman et al. (2012), there is no clear understanding of the origin of chronic back pain in 98% of patients [60]. Moreover, current prevention and treatment methods are open to improvement [61–63]. There is evidence that factors such as high-stress levels, depressed mood, and anxiety are strong predictors of back pain [62, 64, 65]. The recommendations for the treatment of non-specific chronic back pain are based on a biopsychosocial approach promoting the patients’ ability to resume their activities, social interactions, and physical exercise, and it is useful to include psychological treatment for people with persistent symptoms [62, 66]. Research suggests that the highest ratios of reported perceived pain are related to emotional stress [66]. Even the use of opiates (remifentanil) can be blocked by negative patient expectations and be 200% more effective if the patient’s expectations are positive [67]. The effects of a positive expectation were associated with activity in the endogenous pain modulation system and the negative effects of expectation with activity in the hippocampus, results that make us consider that part of the solution to the great problem we are dealing with is the inclusion of a multidisciplinary approach to the patient with chronic back pain. Increased pain management strategies, increasing acceptance of pain, may be the cause of a greater disposition to be involved in daily activities and the encouragement of physical exercise. The results suggest that the intervention could help patients to improve their pain acceptance (CPAQ). The patients in the study had no experience with mindfulness-based practices, a condition we used to evaluate the potential application of mindfulness-based programs to real clinical settings, where very few people would likely have practiced mindfulness. The design was also intended to avoid possible contaminating effects on motivation and adherence. The results show that the program could be used to promote activity (SF-36 physical functioning, role limitations due to physical health), or to increase social interactions (SF-36 social functioning). According to the review by Maher et al. [68], physical exercise, has high evidence as a treatment within non-pharmacological therapies. It is common to advise the patient to practice physical exercise or lead an active life. However, catastrophizing, rumination, or symptomatology of stress, depression, or anxiety could be factors that curb healthy behaviors and thus prolong the patient’s symptomatology. It is necessary to design techniques to increase pain tolerance and psychological distress. MBSR-CBP could be a possible option not only because of its effect, but also because of its potential for adaptability, as it can be applied in large groups, or even in an online format [69]. In this regard, Herman et al. have found that MBSR has been cost saving when compared with usual care alone for chronic lower back pain [23]. The MBSR-CBP of the current study has included psychoeducational contents on pain, the role of psychological distress in the development of pain and the use of effective coping strategies, with a focus on tolerance and acceptance. Ashar et al. have shown that pain reprocessing therapy has been effective in producing substantial and durable pain relief [70]. The focus of this therapy has been the reconceptualization of pain as related to nondangerous brain activity rather than injury in the peripheral tissue. Possible mechanisms could be related on one hand to less response to low back pain in the anterior midcingulate, the anterior prefrontal cortex, and the anterior insula for PRT vs usual care [70]. In the other hand, it could be related to increase resting connectivity between the primary somatosensory cortex, and the anterior prefrontal cortex and the anterior insula. Furthermore, an increase in the connectivity between the precuneus and the anterior midcingulate [70].

Taking RCTs from the report on noninvasive treatments for chronic pain by Skelly et al. [71], there are two previous papers like the present study using MBSR for the treatment of chronic back pain. Morone et al. demonstrated that MBSR, in conjunction with an active healthy aging program, was effective in reducing self-reported pain disability at post-treatment [20]. These pain-related benefits were not maintained at the 6-month follow-up evaluation. However, Cherkin et al. demonstrated that MBSR reduced functional limitations due to pain among participants with chronic back pain at 4- and 13-month follow-up compared to the standard treatment [22]. Clinically significant improvement in pain management was achieved in 61% of patients compared to 44% of the usual treatment for back pain. In both protocols, no substantial changes were specified concerning the original MBSR. The present study has a smaller sample size, but we have included a measure of cortisol, IL-1β, IL-6, IL-17, and TNF-α exploring a possible biological mechanism that could explain the decrease in depressive symptomatology by decreasing IL-1β. It has been observed that an improvement in sleep quality that is part of an overall improvement in mental health (decreased stress and depression) and an increase in well-being and life satisfaction. Like the studies by Cherkin et al. and Morone et al. [20, 22], we have observed statistically significant changes in disability and perceived pain scales. Morone et al. have shown that MBSR has produced a significant improvement in the CPAQ, Activities Engagement subscale and SF-36 Physical Function with 3 months of follow-up [21]. Cherkin et al. have shown that MBSR has achieved a clinically significant improvement in the RDQ (60.5% for the MBSR group vs. 44.1% for the usual care group) and in pain bothersomeness (43.6% for the MBSR group vs. 26.6% in the usual care group) [22]. The effects of the MBSR have been durable with little changes over 13 months. However, Morone et al. have found that the effects of mind-body program for chronic low back pain have improved short-term function and long-term current and most severe pain [20]. But the functional improvement has not been durable (follow-up time has been 6 months). Worth to mention, the study by Morone et al. has recruited older patients (≥ 65 years).

The study did not include post-treatment measures, which is a limitation of the present study. Although patients and clinician were not blinded to the assigned group. The statistical analysis was performed blindly. Although the measurement of the allostatic load has not been included, the study has assessed the negative affective states in patients with low back pain and degenerative disk disease. Data on the usual therapy has not been collected and differences between patients/centre could not be excluded. However, all participants have been asked not to change their clinical habits or initiate new pharmacological or clinical treatments. Since there has been no active control group, benefits cannot be attributed specifically to mindfulness practice. Benefits may be compounded by factors like group support, teacher support, expectations and attention received. In addition, a mindfulness-based program is composed of many elements, apart from the practice itself, like pain pedagogy, lifestyle pedagogy and its impact on health, and body and stretching practices. Specific designs are needed to dismantle the contributing factors. The sample size is not large enough to generalize the results, yet the statistics are sufficiently robust to show an appreciable trend of improvement in the experimental group. Experimental designs with a larger sample size are needed to generalize the results.

## Conclusions

MBSR-CBP could remediate the negative impact of chronic low back pain on the inflammatory, psychological and functional domains. Patients who performed the program showed decrease in depression, stress, perceived pain, disability, and limitations related to health. It also increased the physical function, sleep quality, life satisfaction, well-being and vitality. In the coping with pain domain, the MBSR-CBR increased pain acceptance, self-compassion and significantly reduced the overidentification, kinesiophobia and catastrophization. It also decreased the blood level of IL-1β.

## Supporting information

S1 File Supplementary file describes in detail the exercises that had been implemented in this study. (PDF)

S2 File Supplementary file describes in detail the variables that had been measures in this study. (XLSX)

S3 File (PDF)

S4 File (PDF)

S5 File (PDF)

S1 Checklist (PDF)
