# Correction: Confronting historical legacies of biological anthropology in South Africa—Restitution, redress and community-centered science: The Sutherland Nine

**DOI:** 10.1371/journal.pone.0325596

**Published:** 2025-05-27

**Authors:** Victoria E. Gibbon, Loretta Feris, Joscha Gretzinger, Kathryn Smith, Simon Hall, Nigel Penn, Tinashe E. M. Mutsvangwa, Michaela Heale, Devin A. Finaughty, Yvonne W. Karanja, Jan Esterhuyse, Daniël Kotze, Nina Barnes, Geney Gunston, Je’nine May, Johannes Krause, Caroline M. Wilkinson, Stephan Schiffels, Doreen Februarie, Sianne Alves, Judith C. Sealy

The name Igue We appears incorrectly throughout the article. The correct name is |gui.

In Figs 2 and 4 and Table 1, the name “Igue We” should have been “|gui”. Please see the correct Figs 2 and 4 and Table 1 here.

**Fig 2. Facial reconstructions presented as 2.5D stills from frontal screenshots of 3D models.**

Photographic textures applied using digital montage techniques.

**Fig 4. δ** ^**15**^ **N plotted against δ** ^**13**^ **C for bone and dentine samples analysed in this study.**

Points labelled 1 and 2 represent dentine from Cornelius’s first incisor crown (1 is the occlusal half of the crown, 2 is the half closest to the cemento-enamel junction) and point 3 is the root tip of his mandibular second molar. Point 4 indicates dentine from the occlusal half of Saartje’s first incisor crown.

There is an error in the caption of S4, S7, S8 and S9 Figs. Please see the complete, correct S4, S7, S8 and S9 Figs caption here.

S1 File is incorrect. Please view the correct S1 File below.

S1 Table is omitted from the list of Supporting Information. It can be viewed below.

**Supporting Information**

**S4 Fig. Digital 3D models illustrating cranial trauma.**

Top is Klaas’s cranium, illustrating the perimortem trauma. Left anterolateral view, shows entrance wound on the anterior surface of the left maxilla inferior to the orbit and medial to the left infraorbital foramen. A radiating fracture extends through the left lacrimal, ethmoid and sphenoid bones. Top right shows the inferior view, with a radiating base-of-skull fracture through the occipital bone on the left side of the foramen magnum. The bottom images are of |gui’s cranium illustrating the perimortem trauma. Bottom left, left superolateral view, radiating fractures from the points of impact on the right are observed dissipating through to the sagittal and coronal sutures; a portion of this energy dissipates into the left parietal bone with production of a primary radiating fracture extending posteriorly, which terminates inferior to the left eurion. Bottom right, two points of impact are observed with radiating fractures.

**S7 Fig. Process of mandible estimation for |gui (left, showing in-filled cranial cavity), Voetje (middle) and Klaas (right).**

**S8 Fig. 3D cranial model of |gui from CT data (left) and reconstructed parts (right) including realigned parietal bones and teeth lost post-mortem.**

**S9 Fig. Process of facial reconstruction (left, middle) and final depiction (right) for |gui.**

**S1 File.**

**S1 Table. Consultation guidelines for restitution and repatriation processes covered legally under the National Heritage Resources Act (Act 25 of 1999) (NHRA) and Promotion of Administrative Justice Act (Act 3 of 2000) (PAJA).**

**S1 Dataset. Genetic analyses sample statistics overview used reference data, mitochondrial DNA and Y chromosome haplogroup determination, and qpAdm modelling.**
