# Supplementary material for: The effect of mindfulness on the inflammatory, psychological and biomechanical domains of adult patients with low back pain: A randomized controlled clinical trial
Source: PLoS One. 2022 Nov 9;17(11):e0276734. doi: 10.1371/journal.pone.0276734 (PMC9645607; doi:10.1371/journal.pone.0276734)
Supplement: S1 File — (PDF) [file pone.0276734.s001.pdf]

Mindfulness Based Stress Reduction for Chronic Back Pain

June 14, 2022

Contents

1 Protocol **2**

1.1 Bodywork Sequences . . . 3

1.1.1 Bodywork in lying down postures . . . 3

1.2 Breathing exercises . . . 11

1.2.1 decongesting the rib cage by developing the breathing gesture . . . 11

1.2.2 Diaphragmatic strengthening and distention . . . 11

1.2.3 Mobilization of the intercostal muscles . . . 12

1.2.4 Complete breathing . . . 12

1.3 Posture sequence to prepare spine extension . . . 13

1.4 Toning and relaxation of the postural musculature in standing position . . . 22

1 Protocol

The MBSR programme is a psychoeducational programme founded by Jon Kabat-Zinn. It lasts 31 hours, divided into an introductory session of 1 hour, 6 sessions of 2.5 hours, 2 sessions - the first and the last - of 3 hours, plus an intensive day of practice of 8 hours. The sessions explain formal practices that are intended to be practised daily, informal practices that aim to incorporate mindfulness into everyday actions, and theory on the mechanisms of action of mindfulness to regulate stress and psychological suffering.This intervention is based on the curriculum published by Santorelli et al. [2017] and we have included some minor elements to adapt it to a population with chronic back pain. Instead of using the original MBSR posture sequences, we designed a set of sequences that, under the supervision of physiotherapy professionals, seemed more suitable for people to practice at home, minimising injury. Body work is always accompanied by mindfulness. Participants are invited to attend to the sensations of the body, both pleasant and unpleasant, with acceptance and self-care. Some narratives that were introduced in the body postures guide were (adapted from [O'SULLIVAN and Lin, 2014]):

Promoting a biopsychosocial approach to pain.

- Your back can be in pain from awkward movements and postures, muscle tension, inactivity, lack of sleep, stress, worry and low mood.
- Getting good sleep, exercise, a healthy diet and reducing unhealthy habits (smoking, excessive drinking, etc.) will help your back too.
- The brain acts as an amplifier, the more you worry and think, with catastrophic thoughts, the more pain increases.

Promoting resilience

- Your back is one of the strongest structures in the body, learn how to use it with precision.
- There are many other things you can pay attention to. It's not all about pain.

- If you act with care, exploring the best use of your body in each movement, it is very rare to hurt your back.
- Body awareness is a great antidote to back pain, as it helps to improve precision in movement.

Encourage normal activity and movement

- Relaxed movement will help your back pain subside
- Your back gets stronger with movement
- Movement helps to rejuvenate poorly vascularised tissues, such as hyaline cartilage.
- Protecting your back is not about avoiding movement, it is about learning to use your body well.

Concerns about imaging results (MRI) and pain

- MRI changes are normal, such as grey hair.
- Pain does not mean that you are doing harm, but that your back is
- Sensitive
- Movements will be painful at first, like a sprained ankle, but will get better as they are activated

Listed below are the different sequences that were introduced in MBSR:

1.1 Bodywork Sequences

1.1.1 Bodywork in lying down postures

Time = 40 minutes Resources = audio guide

- Semi-supine posture.

  - Hip rotation.
  - Static flexion of the hips.
  - Stretching of the two sides.
  - Flexion of the cervical area.

- Lumbar flexion-extensions from the semi-supine posture.
- Extension posture from the semisupine posture.
  - Dynamic exercise.
  - Establishment in the posture.
- Rotational posture from semi-supine posture.
  - Circumvolutions of the shoulder from lateral posture.
  - Establishment in rotational posture.
- Sequence of postures for the lower extremities.
- Lateral flexion with costal breathing.
- Slight rotation of the dorsolumbar region and stretching of the external aspect of the hip.
- Stretching of the posterior great chain on the wall.
- Stretch of the abductor chain of the hip from the position of stretching the posterior great chain on the wall.
- Stretch of the lumbar region by resting the soles of the feet on the wall.
- Stretching of the neck extensor chain with the legs resting on the wall.
- Chest opening from the posture of stretching the posterior chain on the wall.
- The alignment posture

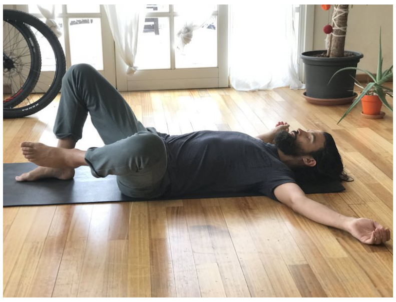

(a) Hips rotation

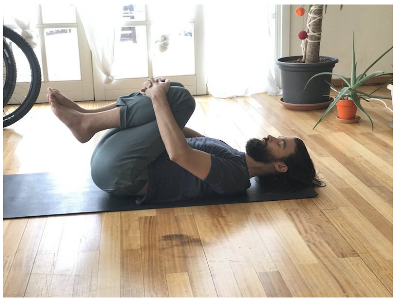

(b) Hip flexion.

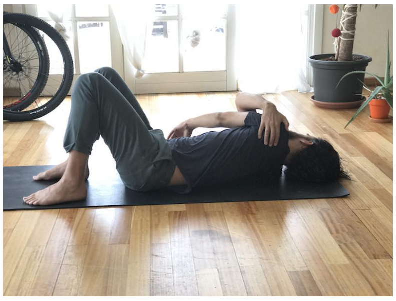

(c) Dorsal region elongation

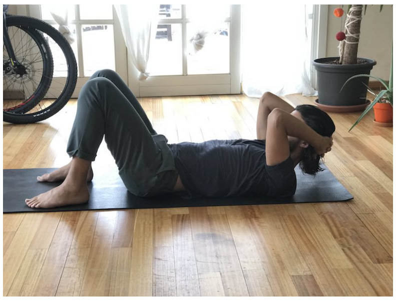

(d) cervical flexion

Figure 2: semisupine stance preparation posture sequences

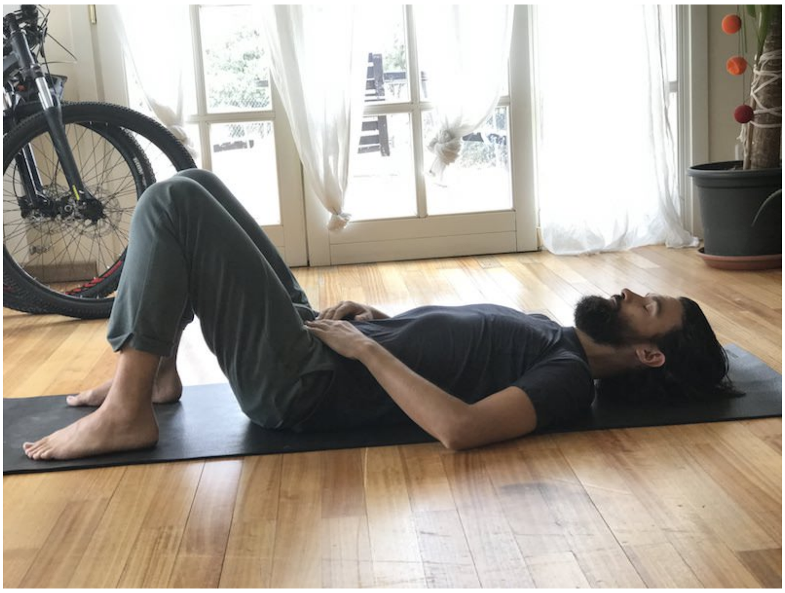

Figure 1: **Semi-supine posture** the following 4 postures show a sequence of postures that are intended to elongate as much as possible the three spinal regions for the establishment of the static posture in unloading.

H

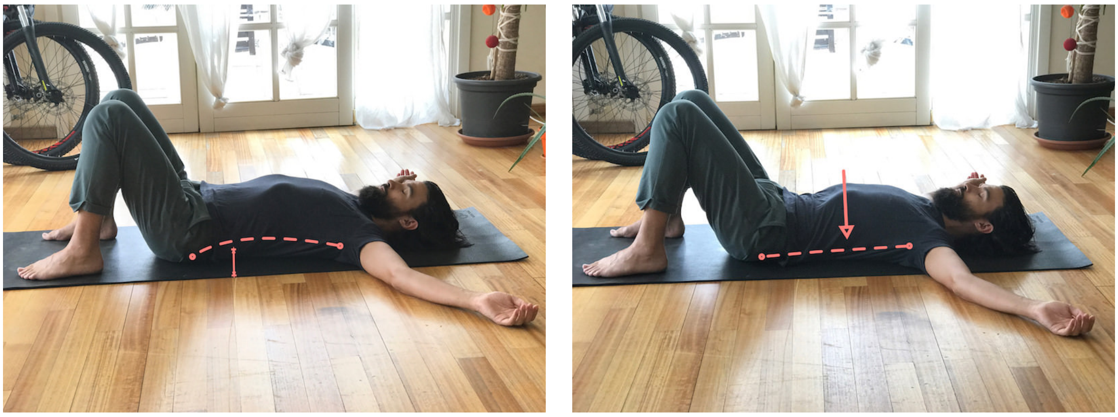

Figure 3: Flexo-extension of the lumbar region. Slow and wide movement.

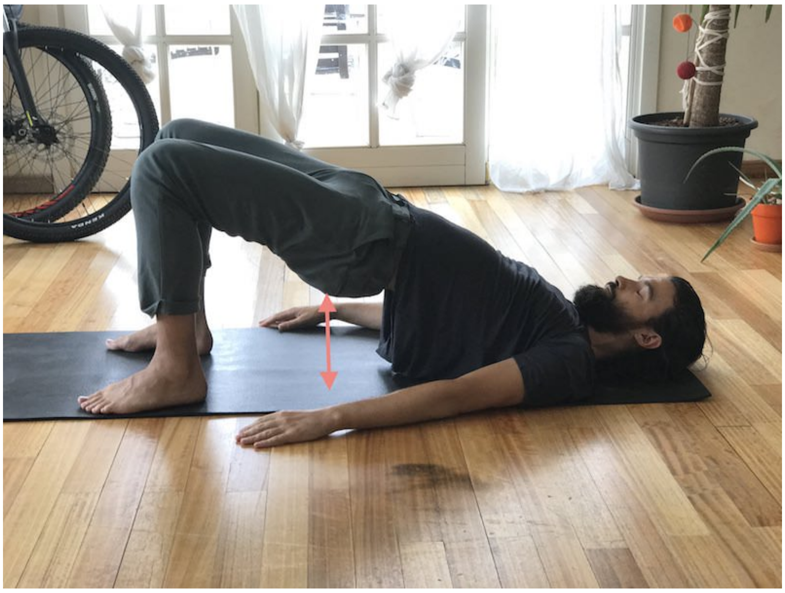

Figure 4: **Column extension.** In the first phase, the upward and downward movement is performed. In phase 2 the static posture is established.

H

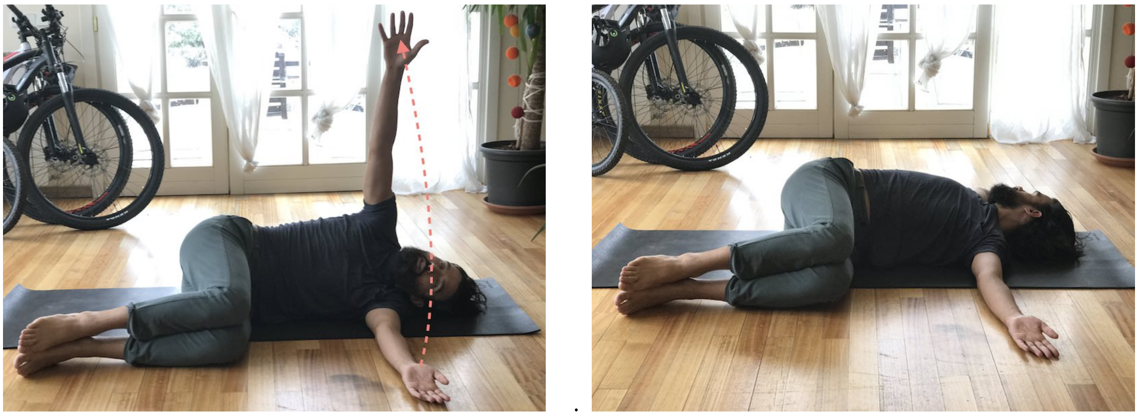

Figure 5: Spine rotation. Phase 1 is dynamic, in phase 2 the static posture is established

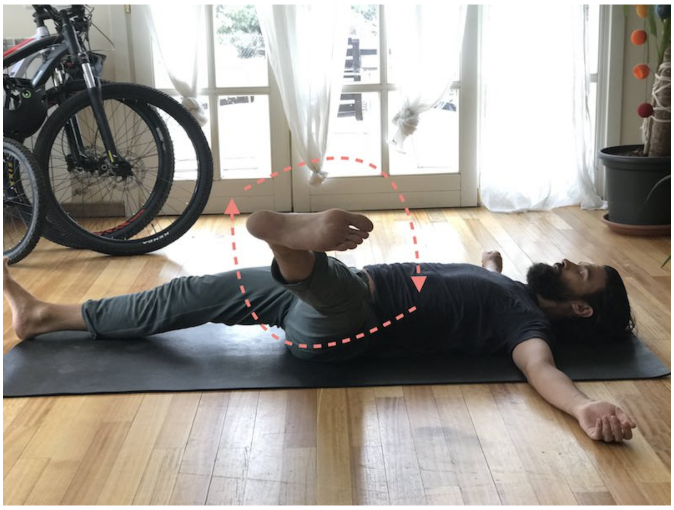

Figure 6: **Rotation of the hips**. With the leg extended, the aim is to make circumvolutions with the hips by dynamically traversing circumferences with increasingly larger radius

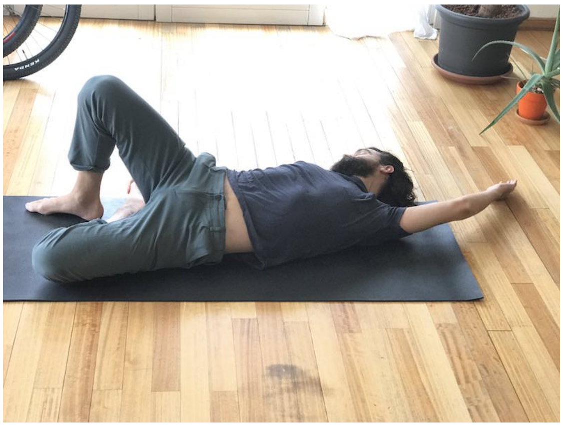

Figure 7: **Lateral flexion from semi-supine stance**. Doing a lateral flexion from the semi-supine posture, the left foot is grasped with the right hand. Breathe into the costal region, fully developing the exhalation in order to elongate the inspiratory muscles. Repeat on the contralateral side.

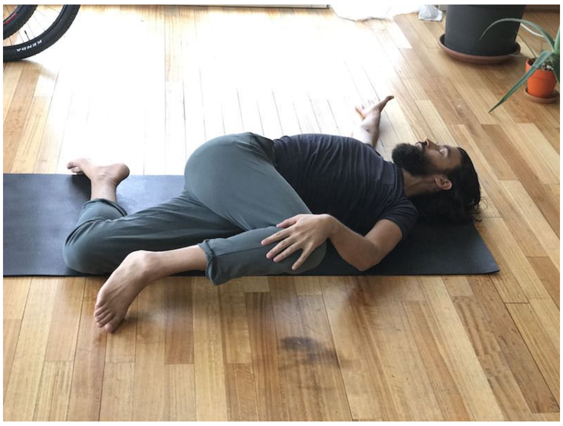

Figure 8: **Slight rotation of the dorsolumbar region and stretching of the external aspect of the hip.** The knee can be brought closer to the floor to graduate the intensity required in the posture.

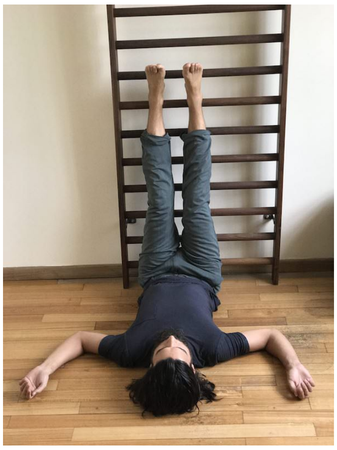

(a) stretching of the large posterior chain

.

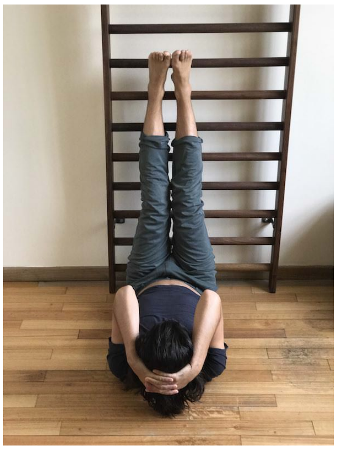

(b) Cervical extensor region elongation

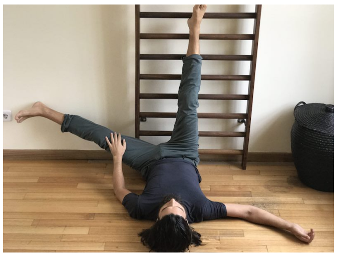

(c) adductor hip chain stretch

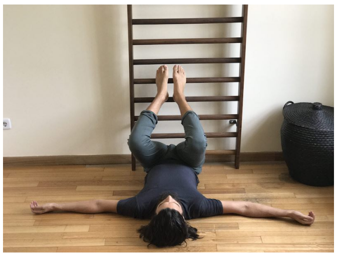

(d) Lumbar stretch and hip flexion.

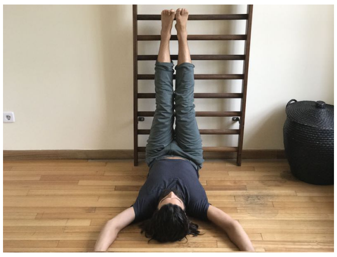

(e) Chest opening supporting exhalation

Figure 9: Sequences of postures on the wall. Tension release is encouraged with the help of exhalation.

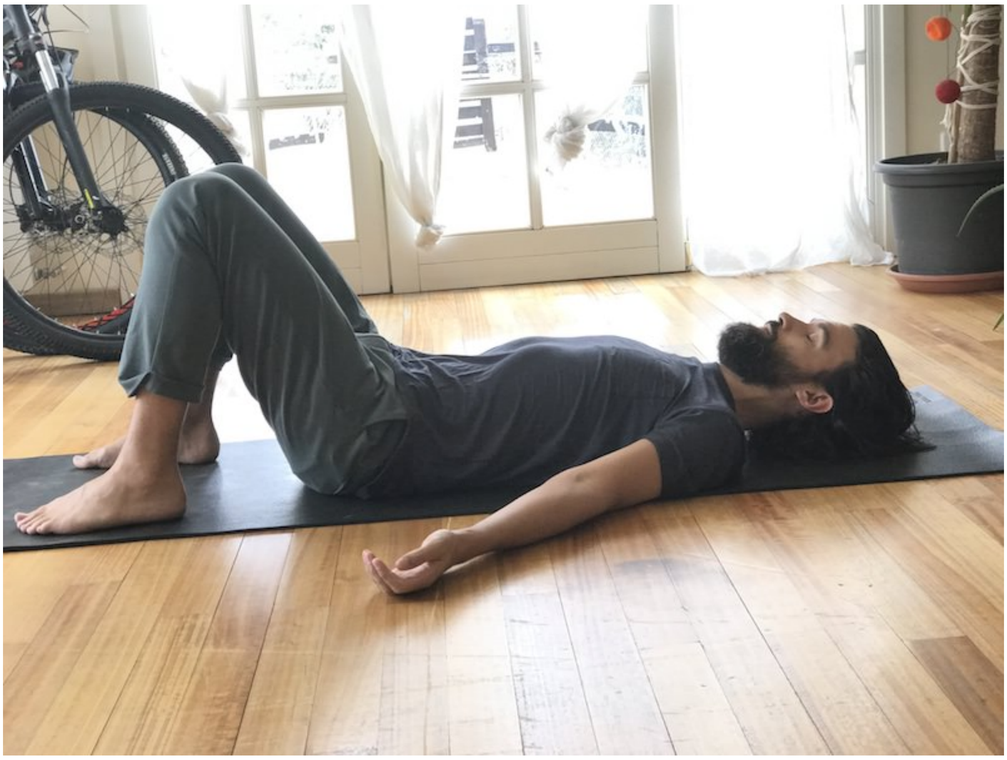

Figure 10: **alignment posture**. Body scan exercise is performed for a few minutes, deepening the sensations of the body.

1.2 Breathing exercises

Time = 40 minutes Resources = audio guide and supplementary readings

1.2.1 decongesting the rib cage by developing the breathing gesture

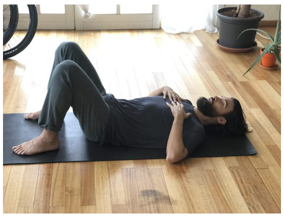

Figure 11: detail of the hands in contact with the upper part of the chest

.

Phase 1. In a comfortable position, preferably semi-supine, the hands are placed on the chest to more easily notice the chest movement on exhalation. The respiratory gesture is different when the body is experiencing distress and one of the most notable characteristics is that the exhalation does not fully develop. There seems to be a spasm in the inspiratory musculature that does not allow the chest to relax during expiration. In this phase it is a matter of carefully following the expiratory gesture detecting the tension patterns in the chest in order to be able to gradually exhale more deeply. It is not a matter of tensing the expiratory muscles, but of relaxing the inspiratory muscles each time the air comes out. The hands can put weight on the chest to help release the tension.

Phase 2. As the practitioner becomes sensitive enough to detect tensions that prevent him/her from breathing out fully, the position of the hands is dispensable. This way of breathing can be developed in the movements of series 1, 3 and 4 and also during everyday life, as it is a good *biofeedback* for knowing when stress is developing.

1.2.2 Diaphragmatic strengthening and distention

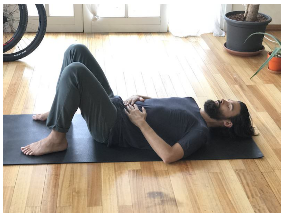

Figure 12: detail of the hands in contact with the belly.

Phase 1. In the semi-supine position, the hands are placed on the belly to more clearly note the abdominal breathing movement. Diaphragmatic dysfunction is often found in patients with back pain and they are unable to breathe by mobilizing the belly. With the help

of images, the primary musculature of breathing is presented: diaphragm, intercostals, serratus major, pectorals and scalenes, underlining the fundamental role of the diaphragm. With the help of a video, the movement of the diaphragm is shown and how it increases and decreases the abdominal pressure, therefore, in inspiration the belly bulges and in exhalation it relaxes. Practitioners are encouraged to raise their hands (which are in contact with the belly) with the help of inspiration and to relax the belly by noticing how they lower their hands on exhalation. **Phase 2**. While breathing abdominally, pay attention to the change of pressure in different areas. Practitioners notice how the zones expand on inspiration and relax on exhalation. Breathing is observed in the lumbar area, the lower region of the rib cage and the pelvic base. Finally, abdominal breathing is noted throughout. This breathing also represents another great *biofeedback* since in everyday life, when stress develops the breathing becomes very shallow, rapid and choppy. The abdominal region becomes tense and impedes the development of breathing.

1.2.3 Mobilization of the intercostal muscles

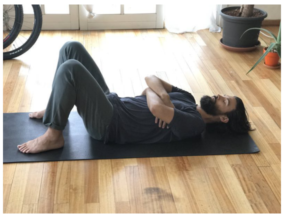

Figure 13: detail of the hands in contact with the belly.

In the semi-supine position, the hands are placed as comfortably as possible on the costal region, so that the movement of the sides can be felt during breathing. Inhale by increasing the distance between the sides and feeling the expansion in the back. Breathing should be done slowly so as not to exhaust the body.

1.2.4 Complete breathing

In semi-supine position, or seated, breaths are taken using all the vital capacity. The inspiration begins with an abdominal phase followed by an intercostal and high phase. It is suggested to the participants to do this breathing slowly and slowly. It would be more advisable, if the execution of the breathing itself requires it, to perform a small retention (expiratory apnea) at the end of the expiratory phase.

1.3 Posture sequence to prepare spine extension

- Semi-supine posture.
  - Hip rotation and static hip flexion.
  - Stretching of both sides.
  - Flexion of the cervical area.
- Extension from semi-supine posture with chest opening.
  - Dynamic extension with dynamic shoulder gyri.
  - Static posture.
- Dynamic rotation with stretching of the limb contralateral to the rotation direction.
- Stretching of the posterior chain with the help of a belt.
- Rotation of the spine with stretching of the posterior chain of the posterior extremity.
- Extension from prone position.
  - Dynamic extensions with neck rotation, hands supported in front.
  - Dynamic extension with contralateral leg lift and rotation.
  - Establishment in extension, elongation of the spine.
- Rotation from prone posture.
  - Dynamically.
  - Establishment in the posture.
- Extension of the lower extremity.
  - Variation of the contralateral posture.

  - Both extremities at the same time.

- Flexion posture, knees and forehead resting on the floor.
- Flexoextension in the posture "on all fours".
- Lateral flexion from the posture on all fours.
- Extension of upper and contralateral lower extremity.
- Squatting position.
  - Dynamic flexo-extension of the knees.
  - Establishment in spinal flexion posture with knees in slight flexion.
- Semi-supine posture.

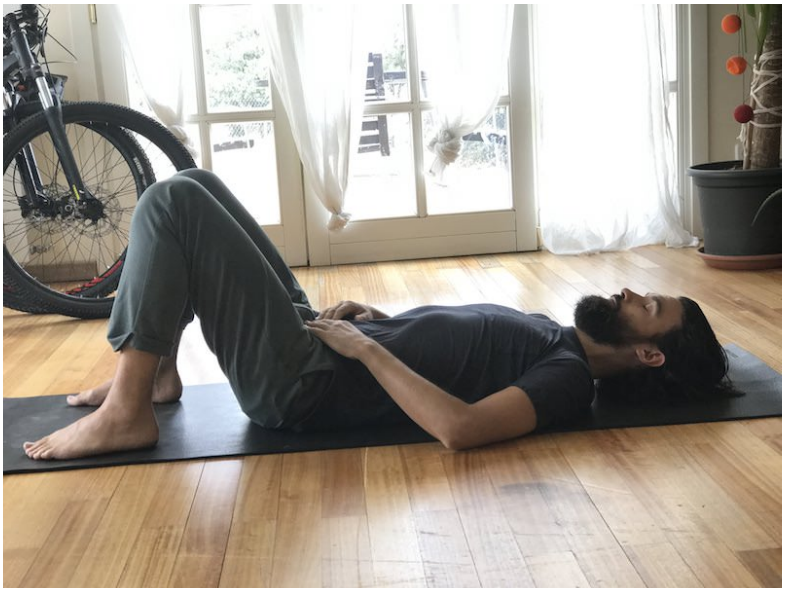

Figure 14: **Semi-supine posture** the following 4 postures show a sequence of postures that are intended to elongate as much as possible the three spinal regions for the establishment of the static posture in unloading.

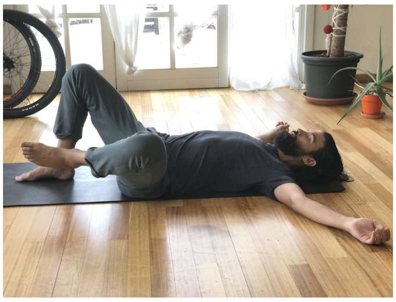

(a) Hips rotation

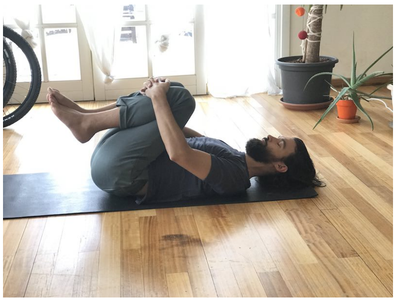

(b) Hip flexion

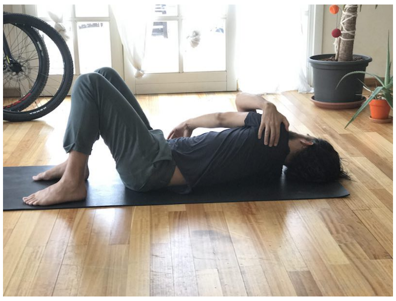

(c) Dorsal region elongation

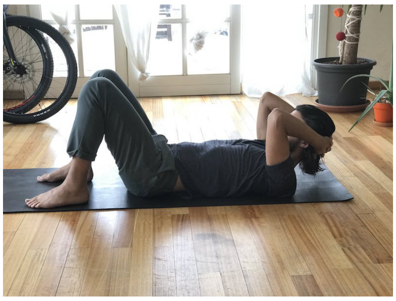

(d) cervical flexion

Figure 15: Semi-supine posture preparation posture sequences

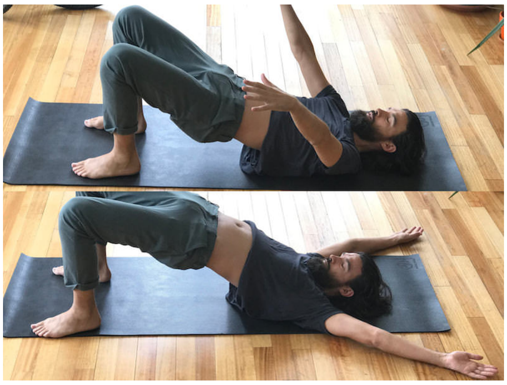

(a) Dynamic Extensions

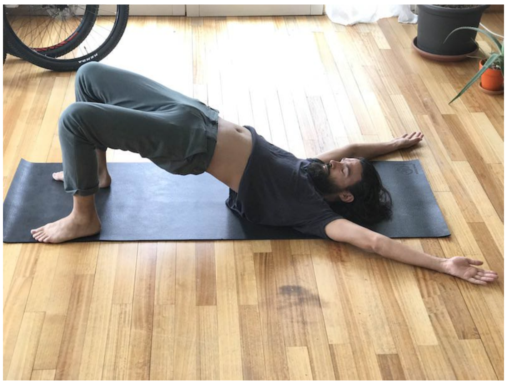

(b) maintenance

Figure 16: Extension sequence from supine posture. Dynamic phase (a) and static phase (b)

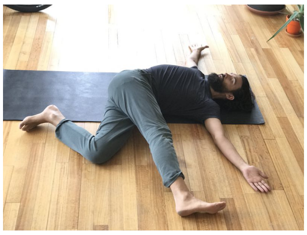

(a) rotation

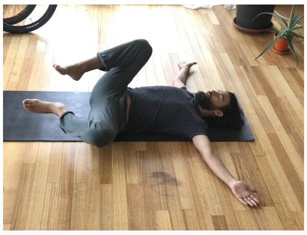

(b) elevation

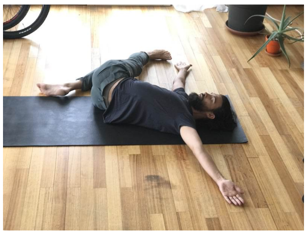

(c) rotation

Figure 17: Spine rotation and stretching of the leg contralateral to the direction of rotation. Performance of the dynamic and static phase.

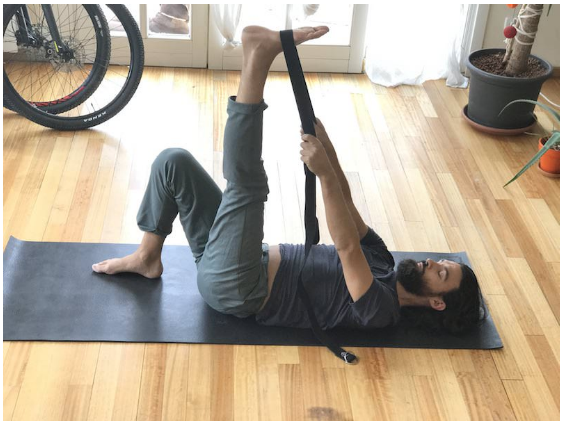

Figure 18: **Belt-assisted posterior chain stretch.** Stretching the arms and maintaining the posture by relaxing the parts involved. Repeat on the contralateral side.

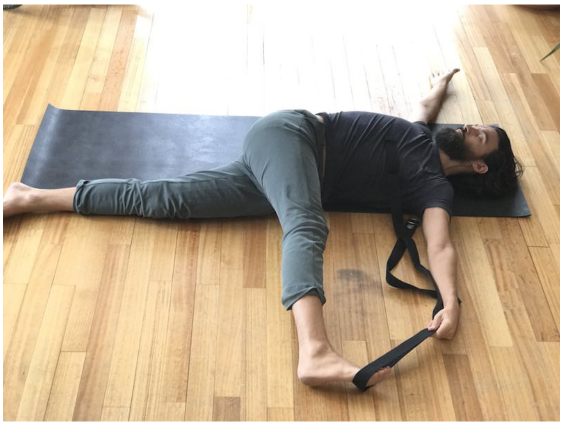

Figure 19: "spine rotation" with stretching of the lower extremity contralateral to the twist

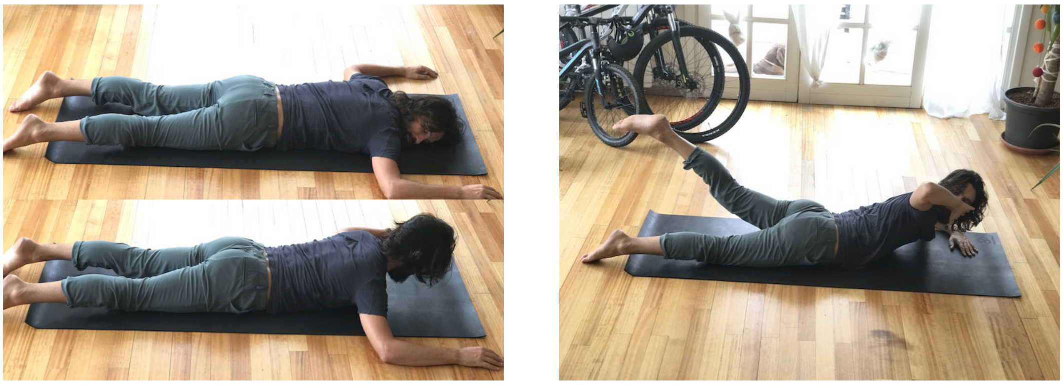

(a) Dynamic Extensions (b) dynamic extensions with slight rotation

Figure 20: Dynamic extensions sequence from prone posture.

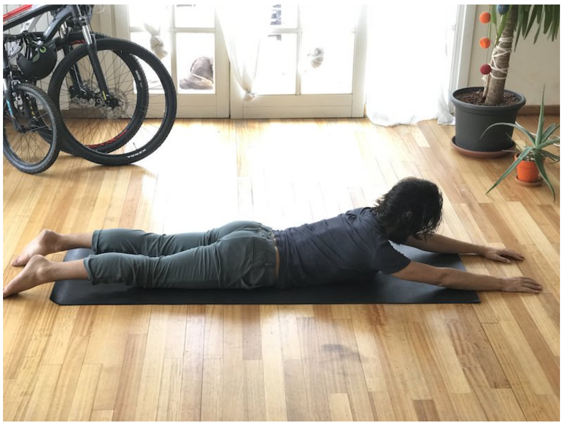

Figure 21: **maintenance of extension posture from prone position** with stretching of the lower extremity contralateral to the twist

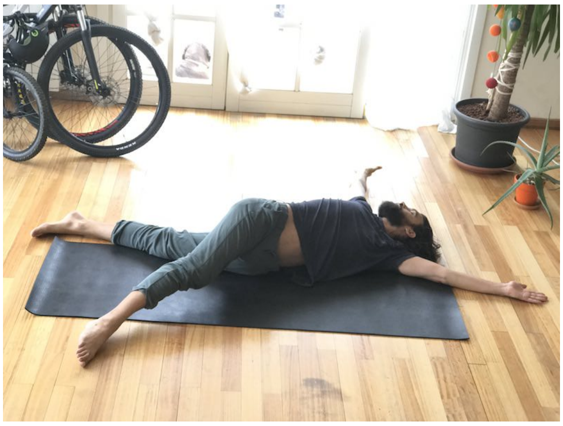

Figure 22: **Spine rotation and chest opening**

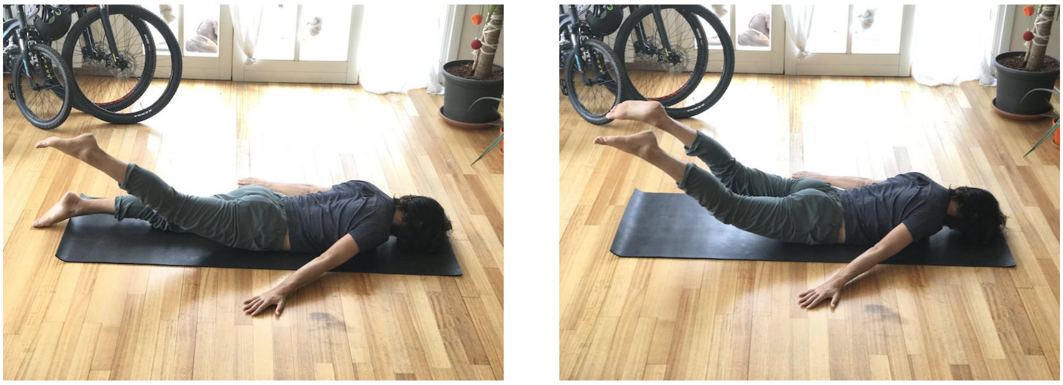

(a) 1 leg elevation (b) elevation of both legs

Figure 23: Lifting sequence of the lower extremities. In phase (a) each leg is lifted separately, in phase (b) both legs are lifted at the same time.

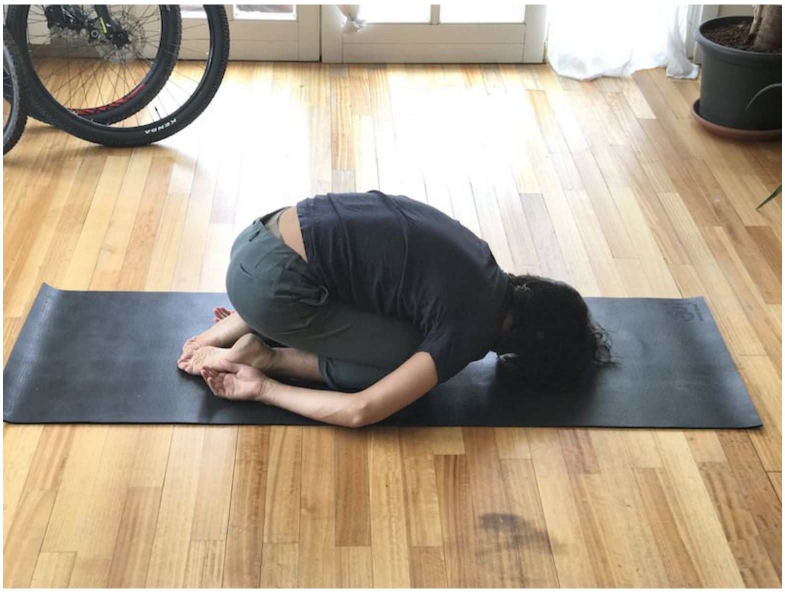

Figure 24: **Lumbar region flexion** with forehead resting on the floor and buttocks on the knees

.

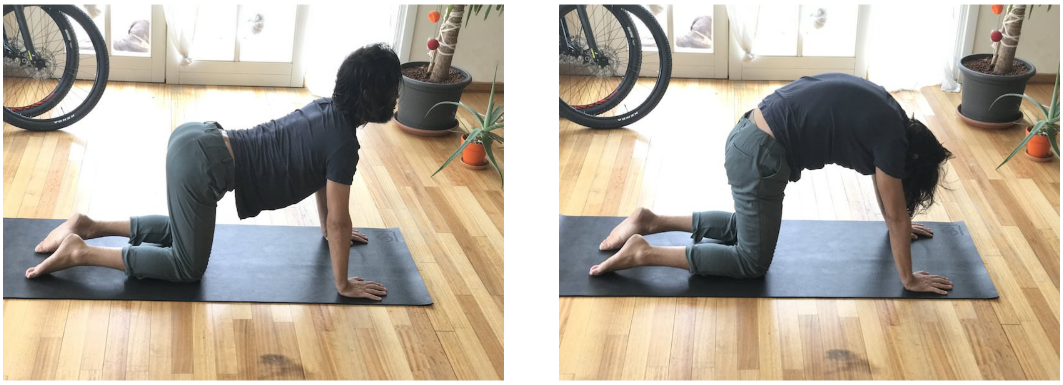

(a) Extension (b) Flexion.

Figure 25: flexo-extension of the spine in the posture *crawl*.

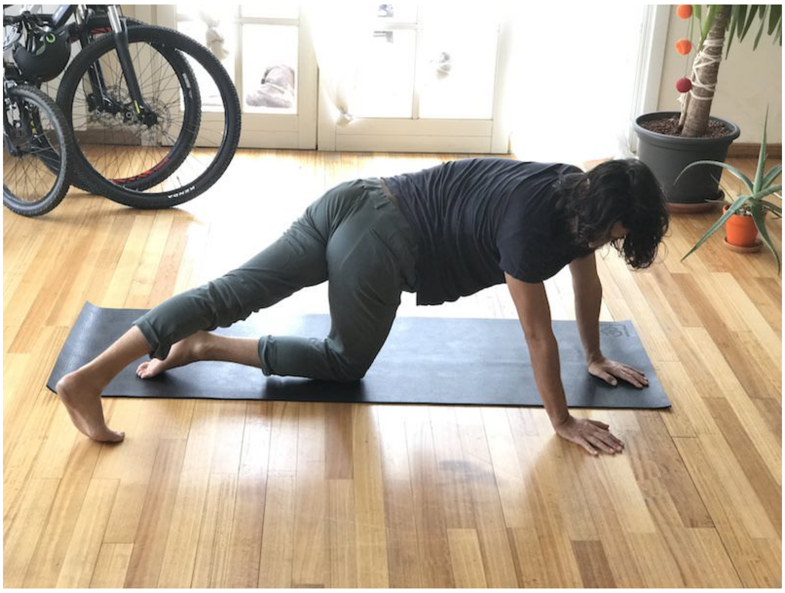

Figure 26: **Lateral spinal flexion** from the posture on the knees. Repeat the posture on the opposite side

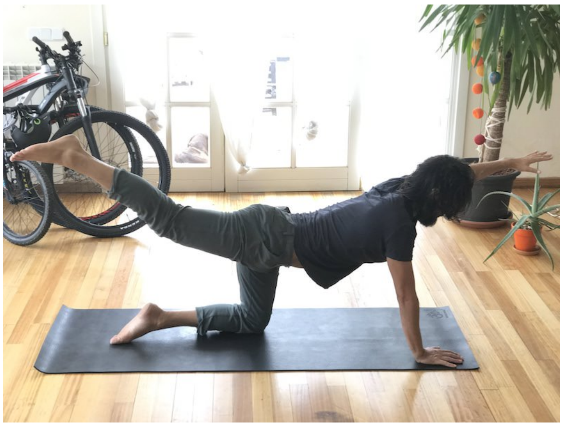

Figure 27: **Balancing with contralateral upper and lower extremity elevation**. It is performed on both sides while maintaining the posture for the appropriate amount of time

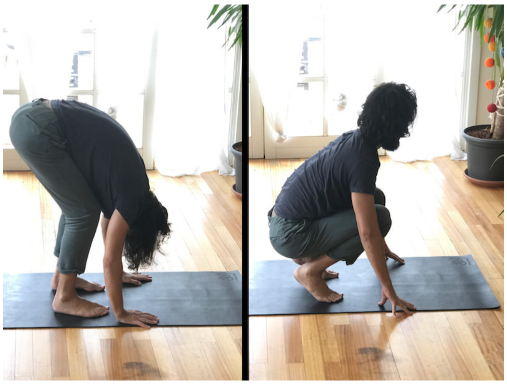

(a) dynamic flexo-extension

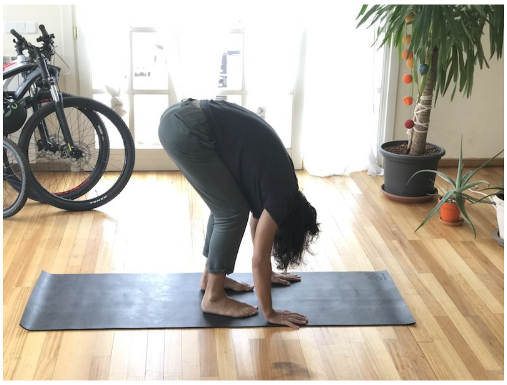

(b) static flexion

Figure 28: Flexion-extension of the hips and knees. In the static phase (b) the knees are to be bent to stretch the back as far as possible.

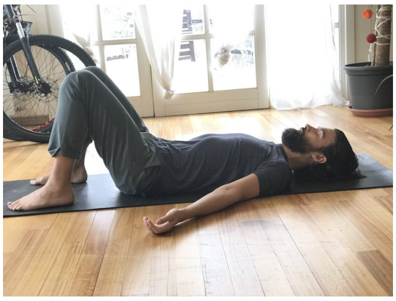

Figure 29: alignment posture

At the end of the sequence it is proposed to perform, in semi-supine posture, a body scanning exercise in 5 minutes.

1.4 Toning and relaxation of the postural musculature in standing position

Time = 40 minutes Resources = audio guide

- Standing posture.
- Circumvolutions of the shoulders in the standing posture.
- Circumvolutions of the cervical region in standing position.
- Mobilization of the shoulders. Anterior rotation, elevation, posterior rotation. Out of phase 180º.
- Elongation of the side, elevation of the upper extremity.
- Lateral flexion in standing position.
- Balance in standing position.
  - Dynamic balance, passing from one foot to the other.
  - Plant of the foot in contact with the contralateral instep, arms parallel to the floor.
  - Balance on one foot, raising the contralateral leg.
- Strengthening of the lower extremity.
  - Knee flexo-extension with chest opening and closing. Upright spine.
  - Knee flexion in standing position with the back leaning against the wall.
- Hip flexion with leg strengthening (half sun salutation).
- Spine extension.
- Flexion posture of the hips.
- Strengthening of the lower extremity.

  - Standing static posture with knees bent.

- Hip flexion with leg opening.
- Twisting in seated position.
- Elongation of the posterior chain of the leg with flexion of the contralateral extremity.
- Twisting in seated position second variant.
- Alignment posture (from semi-supine posture).

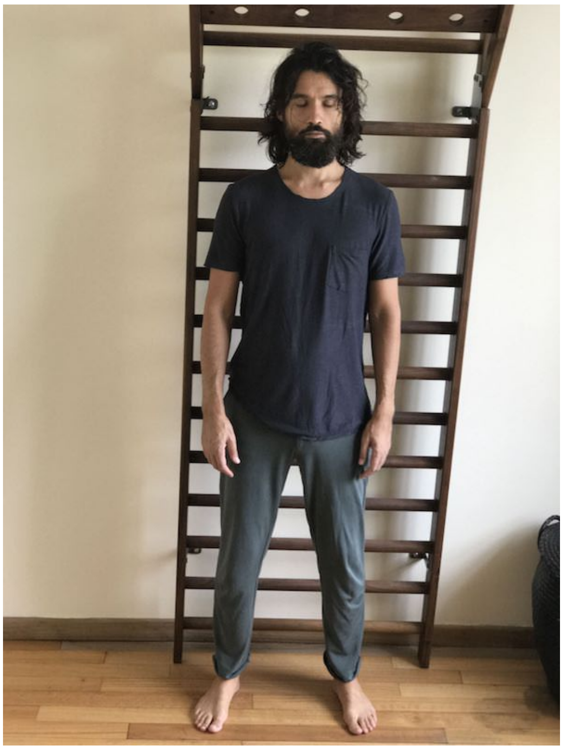

Figure 30: **Standing posture**. A standing body eploration is performed with cues to project weight more effectively to the floor and discover upright posture in the spine.

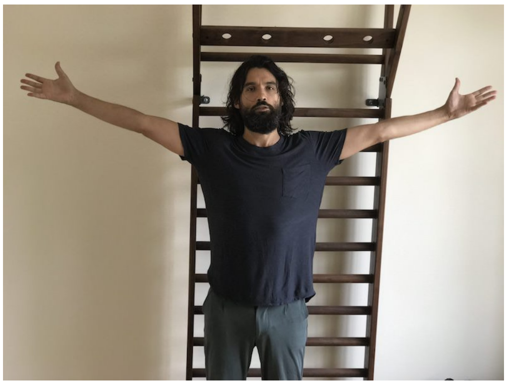

(a) opening

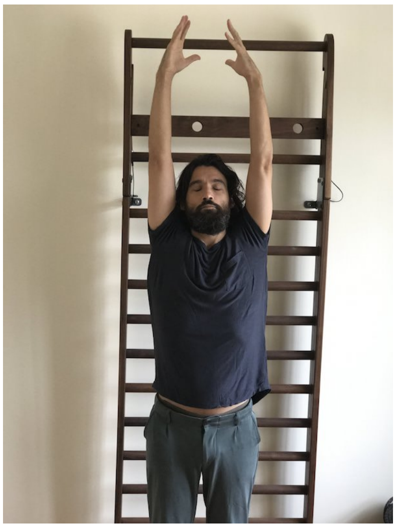

(b) elevation

Figure 31: "Chest opening" with mobilization of the arms. Movement fused with breathing.

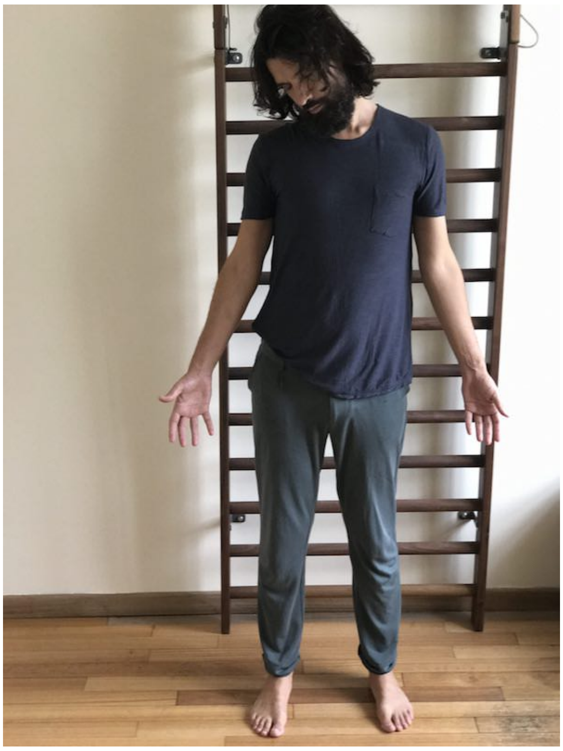

Figure 32: **Circumvolution of the cervical region**in standing position
.

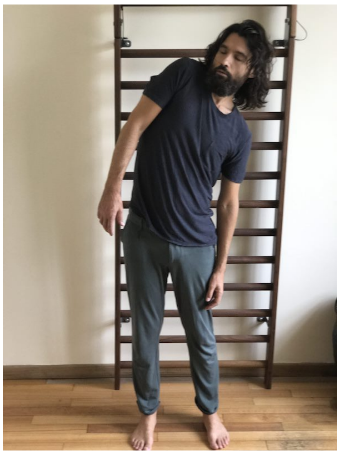

Figure 33: **Shoulder mobilization.** Anterior rotation, elevation, posterior rotation. Offset 180°.

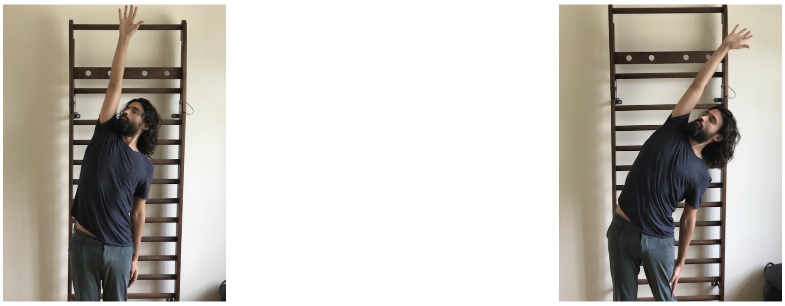

(a) Limb elevation

(b) lateral flexion

Figure 34: Perform once on each side the exercise (a) with the intention of elongating the line from the foot to the hand and then repeat the process generating a lateral flexion, as shown in (b).

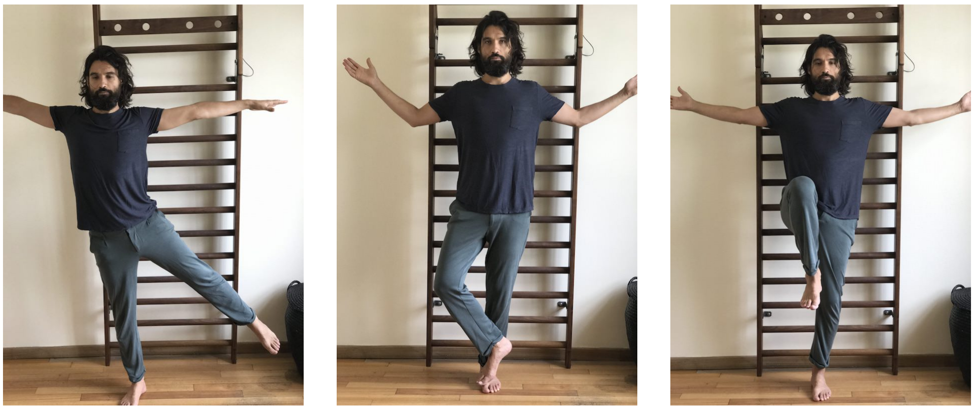

(a) (b) (c)

Figure 35: Sequence of standing balance postures, performed from both sides. In (a) Dynamic balancing, passing from one foot to the other in (a), sole of foot in contact with contralateral instep with arms parallel to the ground in (b) and balancing on one foot, contralateral leg lift

.

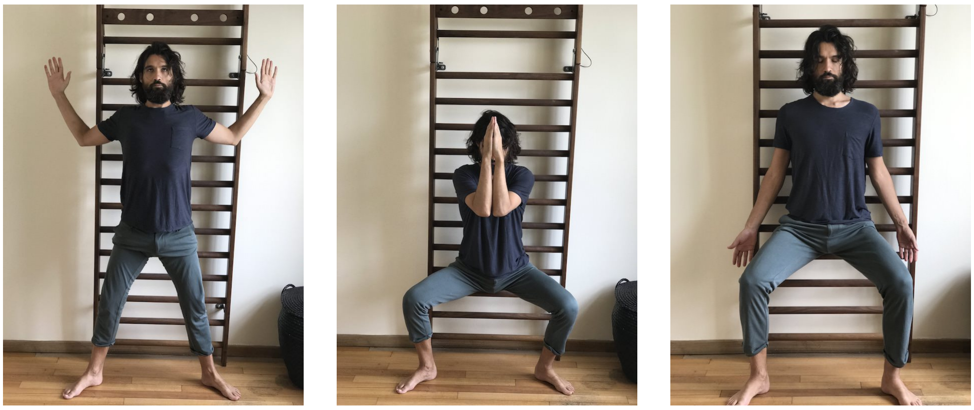

(a) Motion (b) Motion (c) static posture

Figure 36: Strengthening of lower extremity. Firstly dynamic movement (a) and (b) and secondly establishment in the static posture *supporting the back against the wall* (c).

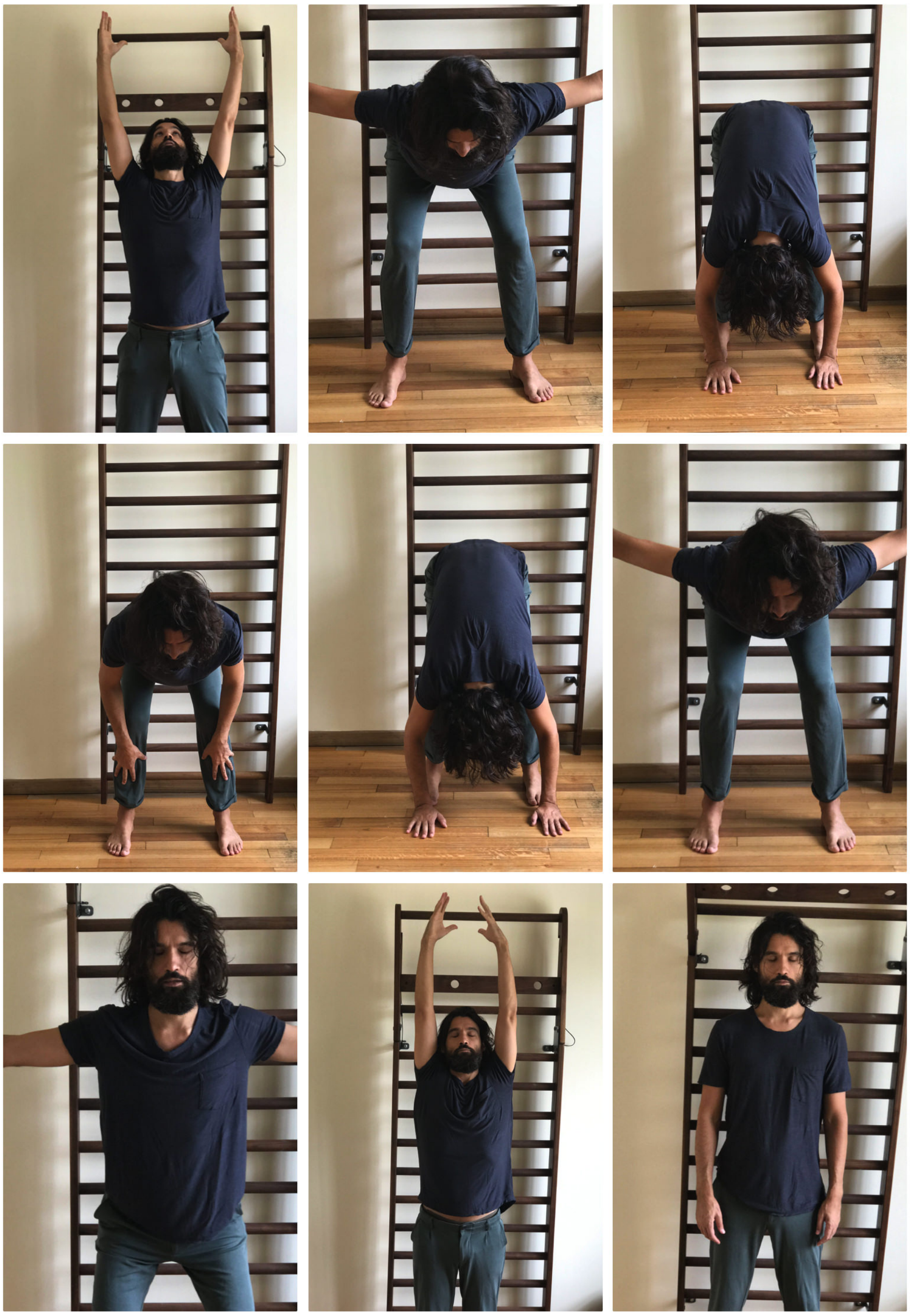

Figure 37: **Dynamic sequence of postures.** Half sun salutation.

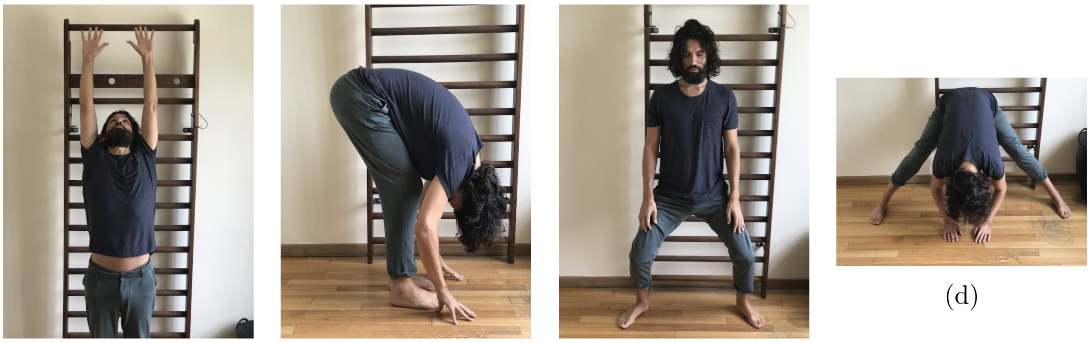

(a) (b) (c) (d)

Figure 38: **Sequence of postures.** In each posture the appropriate amount of time remains . Extension in standing posture in (a), flexion of the hips in standing posture in (b), strengthening of legs, without wall support in (c) and flexion with legs open in standing posture in (d).

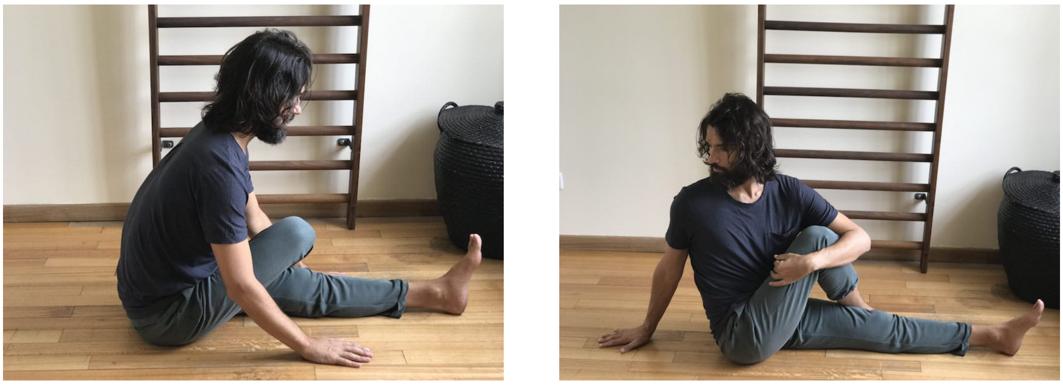

(a) (b)

Figure 39: **Sequence of seated postures.** Elongation of the posterior leg chain with flexion of the contralateral limb in (a) and twisting in seated position in (b).

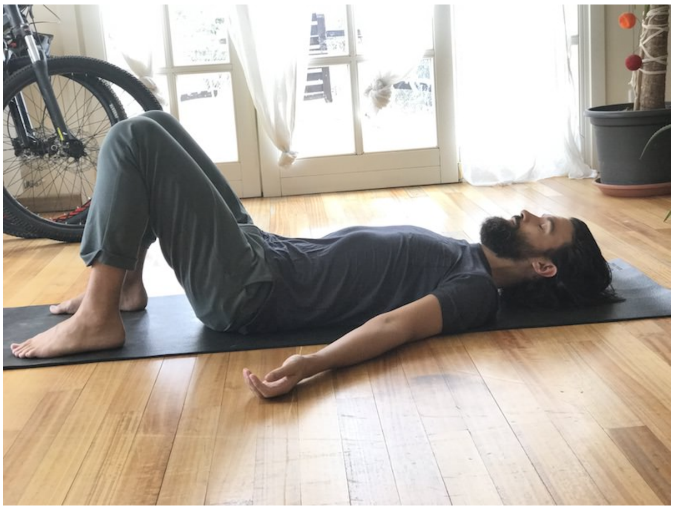

Figure 40: **Semi-supine posture.** Body scan in semi-supine posture.

References

O'SULLIVAN, P. and Lin, I. (2014). Acute low back pain. *PAIN*.

Santorelli, S. F., Meleo Meyer, F., Koerbel, L. K., and Kabat-Zinn, J. (2017). *8-week-mbsr-authorized-curriculum-guide-2017.pdf*. Center for Mindfulness in Medicine, Health Care, and Society (CFM). University of Massachusetts Medical School.
