# Supplementary material for: The effect of mindfulness on the inflammatory, psychological and biomechanical domains of adult patients with low back pain: A randomized controlled clinical trial
Source: PLoS One. 2022 Nov 9;17(11):e0276734. doi: 10.1371/journal.pone.0276734 (PMC9645607; doi:10.1371/journal.pone.0276734)
Supplement: S3 File — (PDF) [file pone.0276734.s003.pdf]

PROTOCOLO DE ENSAYO CLÍNICO

Programa de Reducción del Estrés Basado en Mindfulness para Pacientes con Discopatías

PROMOTOR: Fundación Eduardo Anitua
Instituto Eduardo Anitua
C/José María Cagigal nº19.
Vitoria-Gasteiz
Tfn: 945 16016050

(Fecha y firma)

Investigador Coordinador Gustavo G. Díez.
Instituto Nirakara
Racimos, 10, 28070, Colmenarejo
Madrid

(Fecha y firma)

Investigadores Principales Eduardo Anitua - Fundación Eduardo Anitua
Gustavo G. Díez - Instituto Nirakara
Carmelo Vázquez - Universidad Complutense de Madrid

Índice

1 INFORMACIÓN GENERAL ... 4
1.1 IDENTIFICACIÓN DEL ESTUDIO ... 4
1.2 TIPO DE ESTUDIO CLÍNICO ... 4
1.3 DESCRIPCIÓN DE LOS PRODUCTOS EN ESTUDIO ... 4
1.4 DATOS RELATIVOS AL PROMOTOR ... 4
1.5 MONITORIZACIÓN DEL ESTUDIO ... 4
1.6 CENTROS PARTICIPANTES ... 5
1.7 DATOS DE LOS INVESTIGADORES ... 5
1.8 DURACIÓN PREVISTA DEL ESTUDIO ... 6
2 JUSTIFICACIÓN Y OBJETIVOS ... 6
2.1 JUSTIFICACIÓN ... 6
2.2 OBJETIVOS ... 10
3 TIPO DE ESTUDIO Y DISEÑO ... 11
3.1 TIPO DE ESTUDIO. ... 11
3.2 PROCESO DE ALEATORIZACIÓN ... 11
3.3 TIPO DE CONTROL Y DISEÑO ... 11
3.4 TÉCNICAS DE ENMASCARAMIENTO ... 11
4 POBLACION DE ESTUDIO ... 12
5 PROCEDIMIENTO DE RECLUTAMIENTO DE PACIENTES ... 12
6 SELECCIÓN DE LOS SUJETOS ... 13
6.1 CRITERIOS DE INCLUSIÓN ... 13
6.2 CRITERIOS DE EXCLUSIÓN ... 14
6.3 NÚMERO DE SUJETOS PREVISTOS ... 14
6.4 CRITERIOS DE RETIRADA Y ANÁLISIS DE LOS MISMOS ... 14
7 TRATAMIENTOS DEL ESTUDIO ... 15
7.1 TRATAMIENTOS DEL ESTUDIO ... 15
7.2 TRATAMIENTOS NO PERMITIDOS ... 16
8 VARIABLES DE ESTUDIO ... 16
8.1 VARIABLES DEMOGRÁFICAS: ... 16
8.2 VARIABLES DE RESULTADO: ... 16
8.2.1 PRIMARIAS ... 16
8.2.2 SECUNDARIAS ... 17

8.3 COVARIABLES ... 21
8.4 EVALUACIÓN DE LA RESPUESTA ... 23
La base de datos de cada participante consta de 3 bloques de medidas: Demográficas, psicológicas, y biológicas. ... 23
9 DESARROLLO DEL ESTUDIO-CRONOGRAMA ... 24
9.1 VISITA RECLUTAMIENTO ... 25
9.2 VISITA DIAGNÓSTICA ... 25
9.3 VISITA INCLUSIÓN ... 25
9.4 VISITA TRATAMIENTO ... 25
9.5 VISITA SEGUIMIENTO ... 26
9.6 FINALIZACIÓN DEL ENSAYO CLÍNICO ... 26
10 SEGURIDAD ... 26
11 SISTEMA DE ALMACENAMIENTO Y CONTROL DE ACCESO A LA INFORMACIÓN ... 26
12 DESTINO DE LA MUESTRA DE SANGRE TRAS SU UTILIZACIÓN EN ESTE ENSAYO ... 27
13 CONSIDERACIONES ÉTICAS ... 27
13.1 CONSIDERACIONES GENERALES ... 27
13.2 INFORMACIÓN AL PACIENTE Y CONSENTIMIENTO INFORMADO ... 27
13.3 CONFIDENCIALIDAD DE LOS DATOS ... 28
13.4 PRESUPUESTO DEL ESTUDIO ... 29
13.5 SEGURO ... 29
14 CONSIDERACIONES PRÁCTICAS ... 30
14.1 NORMAS PARA EL PERSONAL AUXILIAR QUE INTERVIENE EN EL ESTUDIO ... 30
14.2 MANEJO, CORRECCIONES Y ARCHIVO DE DATOS ... 30
14.3 CONDICIONES DE PUBLICACIÓN ... 30
15 BIBLIOGRAFÍA ... 31

1 INFORMACIÓN GENERAL

1.1 Identificación del estudio

TÍTULO:

Programa de Reducción del Estrés Basado en Mindfulness para Pacientes con Dolor Crónico de Espalda

CÓDIGO: FIBEA-07-EC/18/MBSR

1.2 Tipo de estudio clínico

Ensayo clínico controlado y randomizado

1.3 Descripción de los productos en estudio

Tratamiento experimental: Programa de intervención basado en Mindfulness para reducir el estrés, MBSR (Mindfulness Based Stress Reduction)

Tratamiento control: Tratamiento usual según diagnóstico.

1.4 Datos relativos al promotor

Fundación Eduardo Anitua
Instituto Eduardo Anitua
C/José María Cagigal nº19.
Vitoria-Gasteiz
Tfn: 945 16016050

1.5 Monitorización del estudio

La monitorización del estudio se realizará por parte del Instituto Nirakara

Instituto Nirakara

Racimos, 10

28070, Colmenarejo (Madrid)

1.6 Centros participantes

1- Fundación Eduardo Anitua

2- Universidad Complutense de Madrid

3- Instituto Nirakara

1.7 Datos de los investigadores

1- Fundación Eduardo Anitua
Dr. Eduardo Anitua
Clínica Dental Eduardo Anitua
C/José María Cagigal nº19.
01007 Vitoria-Gasteiz
Tfno.: 945 16 06 50

2 - Universidad Complutense de Madrid
Dr. Carmelo Vázquez.
Catedrático de Psicopatología
Facultad de Psicología de la Universidad Complutense de Madrid.
Campus de Somosaguas,
28223 Somosaguas, Madrid

3 - Instituto Nirakara
Gustavo G. Díez.
Instituto Nirakara
Racimos, 10, 28070, Colmenarejo
Madrid

1.8 Duración prevista del estudio

Los plazos aproximados son los siguientes:

- Presentación CEIC: Julio 2018
- Fase experimental: Octubre 2018 – Junio 2019
- Informe final: Octubre 2019

2 JUSTIFICACIÓN Y OBJETIVOS

2.1 Justificación

El Barzouhi y col. (2013) realizaron un ensayo clínico aleatorizado con 267 pacientes herniados y con dolor ciático. Estudiaron cambios diferenciales entre pacientes que fueron intervenidos quirúrgicamente y pacientes que no. Al cabo de 1 año se encontró que:

- Había una mayor evidencia, mediante MRI, de discos herniados y compresión del nervio ciático en los pacientes que no fueron sometidos a cirugía.
- Ninguna diferencia significativa en los resultados entre los dos grupos en las siguientes variables: discapacidad[1], recuperación[2] y dolor percibido[3].

Aunque la mayoría de los pacientes con dolor crónico de espalda presenta algún tipo de discopatía, lo contrario no es cierto: en un estudio, ya clásico, Jensen y col. (1994), averiguó que sólo el 36 % de la población sin dolor de espalda tenía los discos intervertebrales en perfecto estado. Sin embargo, no presentaban síntomas. Kendrick y col. (2001) en un estudio aleatorizado con pacientes con dolor de espalda de bajo riesgo, encontró que aquellos que fueron radiografiados reportaban, en el seguimiento, mayor dolor y peor salud en general en comparación con los que no fueron sometidos a una radiografía. Irónicamente, los pacientes radiografiados estaban más satisfechos, en general, con la atención médica.

Parece que la terapia centrada en el paciente puede reportar mejor rendimiento psicosocial, pero para ello necesitamos una metodología matemática que nos permita trabajar con variables categóricamente diferentes, centrado en la estadística individual más que en medias estadísticas y con capacidad de manejar gran cantidad de datos.

Según el Informe Anual del Sistema Nacional de Salud (Ministerio de Sanidad, 2017), el dolor de espalda lumbar representa 17,3 % de los problemas de salud crónicos en nuestro país. Según un estudio de Humbra Mendiola, Carmona, Pena Sagredo, y Ortiz (2002), casi la mitad de la población adulta española padece, al menos, un episodio de dolor lumbar en un período de 6 meses. Además, Cabrera-Leon y Cantero-Braojos (2017) concluía que çomparado con la población sin dolor crónico (nDC), la población con DCD tiene 6 puntos menos en calidad de vida mental y 12 en la física, consume el triple de medicamentos, utiliza casi el doble los servicios sanitarios, y su ausencia laboral prolongada es el triple. **Además, la población con DC no discapacitante (DCnD) no difiere significativamente de aquella nDC en cuanto a las variables de estudio.**

Según la revisión de Haldeman y col. (2012), entre el 1–2 % de personas con dolor de espalda tienen un trastorno grave o sistémico, entre los que se encuentran: trastorno inflamatorio sistémico, infecciones, tumor espinal o fractura espinal. Sin embargo, en el otro 98 % restante no hay una comprensión clara del origen y tratamiento de la sintomatología y los métodos actuales de tratamiento y prevención parecen que son suceptibles de mejora (O'SULLIVAN & Lin, 2014; Mafi, McCarthy, Davis, & Landon, 2013). Hay evidencia de que factores como **los trastornos del sueño, los altos niveles de estrés, el estado de ánimo depresivo y la ansiedad son fuertes predictores del dolor de espalda** (Gatchel, Peng, Peters, Fuchs, & Turk, 2007; Stubbs y col., 2016). La investigación apunta a que los ratios más altos de dolor percibido están relacionados con el estrés emocional, con una limitada consciencia emocional y una limitada expresión y procesamiento de las emociones (Lumley y col., 2011; Nijs y col., 2014). Incluso el uso de opiaceos (remifentanilo) puede ser bloqueado por unas expectativas negativas por parte del paciente y ser un 200% más efectivo si las expectativas del paciente son positivas (Bingel y col., 2011). Los efectos de la expectativa positiva se asociaron con la actividad en el sistema endógeno de modulación del dolor y los efectos negativos de la expectativa con la actividad en el hipocampo.

Con una instrucción cognitiva relativamente sencilla, deCharms y col. (2005) pudieron observar, utilizando un equipo de resonancia magnética funcional en tiempo real, cómo los sujetos **podían controlar la activación de la corteza cingulada anterior, reduciendo así los síntomas del dolor**. Las áreas que normalmente se activan ante estímulos dolorosos son la corteza somatosensorial primaria (S1), corteza somatosensorial secundaria (S2), corteza cingulada anterior (ACC), ínsula, corteza prefrontal (PFC), tálamo y cerebelo (Apkarian, Bushnell, Treede, & Zubieta, 2012).

En situaciones de anticipación al dolor, se activan algunas de las regiones cerebrales nocioceptivas que son determinantes para la experiencia del dolor y la modulación del dolor, incluyendo las cortezas somatosensoriales, PFC y ACC (Porro y col., 2002). Estos hallazgos indican que las regiones cerebrales

reciben aferencia de dolor a través de múltiples vías y que, al menos en regiones cerebrales superiores, no existe una "línea marcada entre la entrada nociceptiva periférica y la representación cortical final de esa entrada (Bushnell, eko, & Low, 2013). Por ello no es difícil llegar a la hipótesis de que el entrenamiento mental puede ser un elemento fundamental en el tratamiento del dolor y el malestar.

Entre los pacientes con dolor crónico de espalda, normalmente hay un elevado grado de identificación con las narrativas catastróficas y un agotamiento generalizado que ha mermado las posibilidades de disponerse positivamente ante el dolor. Los pacientes con discopatías han desarrollado distrés, alterando eventualmente su equilibrio alostático, agotando sus recursos de afrontamiento y desarrollando trastornos de ansiedad o afectivos en diferentes grados. Resulta complicado poder realizar en un tiempo limitado, propio de una consulta, un tratamiento adecuado de reducción del estrés e influencia negativa de las emociones y cogniciones en el dolor.

El estrés es un poderoso desencadenante de la sintomatología del dolor en pacientes con dolor crónico (Schwartz, Slater, & Birchler, 1994). Parece intuitivo pensar que existe una interrelación estrecha entre dolor y el estrés crónico, pero los mecanismos subyacentes todavía no están claros (Abdallah & Geha, 2017). Ante el dolor, la respuesta de estrés haría buscar al individuo una solución ante la situación amenazante": lucha, huída o congelamiento. Cuando el dolor se cronifica y las estrategias de afrontamiento colapsan, se consolida un "çírculo vicioso" de retroalimentación entre el dolor y el estrés que agota poco a poco toda esperanza y sensación de bienestar para el paciente.

Los pacientes con estrés crónico suelen tener altos niveles de catecolaminas (Cevik, Otahbachi, Miller, Bagdure, & Nugent, 2009). El estrés aumenta los niveles de citoquinas proinflamatorias (el factor de necrosis tumoral y la interleucina 1a y 1b) en la periferia y el cerebro (Madrigal y col., 2001). Se han descrito niveles elevados de mediadores inflamatorios en pacientes con patologías discales y estos niveles aumentan con la gravedad de la degeneración (Weiler, Nerlich, Bachmeier, & Boos, 2005). El estrés crónico también puede influir en la apoptosis celular y la degeneración en los discos intervertebrales (Kermani, Hoboubati, Esmaeili-Mahani, & Asadi-Shekaari, 2014).

Al final de los años 70, Jon Kabat-Zinn diseñó un protocolo basado en mindfulness[4]. Los primeros estudios -no aleatorios-demostraron que el MBSR (Mindfulness Based Stress Reduction) fue eficaz para reducir los síntomas del dolor y la dependencia de analgésicos entre los pacientes con dolor crónico antes de la intervención (Kabat-Zinn, 1982). Más recientemente, varios estudios independientes bien controlados han reproducido y desarrollado este primer trabajo. Morone y col. (2016) demostraron que MBSR, en relación con un programa de envejecimiento saludable activo, fue eficaz en la reducción de la discapacidad del dolor auto-reportado en el post-tratamiento en un gran estudio aleatorizado y controlado con pacientes con dolor

crónico de espalda (N = 282). Estos beneficios relacionados con el dolor no se mantuvieron en la evaluación de seguimiento de 6 meses.

En uno de los mayores estudios controlados y aleatorizados de intervención de atención plena hasta la fecha (N = 342), MBSR **redujo las limitaciones funcionales debido al dolor** entre los participantes de dolor crónico de espalda a los 4 y 10 meses de seguimiento, en comparación con el tratamiento usual (Cherkin y col., 2017). Mientras que este estudio mostró que MBSR proporcionó una mejora clínicamente significativa en el manejo del dolor en un mayor porcentaje de participantes en el seguimiento (61 %) en relación con el tratamiento usual para dolor de espalda (44 %), no hubo evidencia en este estudio que MBSR era superior a un programa de Cognitive-Behavioral Therapy (CBT) (58 %).

Por otro lado, hay suficiente evidencia de que MBSR reduce el estrés (Creswell, 2017) bajando los niveles de citoquinas proinflamatorias (Carlson, Speca, Faris, & Patel, 2007) e influyendo también en factores de transcripción celular y expresión génica (Black & Slavich, 2016), el presente programa parte de la estructura en el MBSR y añade algunas prácticas más específicas para el dolor de espalda que pudieran servir para maximizar los resultados.

Este estudio pretende entender el papel mediador del estrés en la sintomatología del paciente con dolor crónico de espalda. Utilizamos MBSR por ser un programa validado y con efectividad en la reducción del estrés. Mediremos el impacto del entrenamiento por la concentración sérica de Citoquinas ((TNF)-, interleuquinas (IL)-1 /, IL-6 e IL-17) y concentración de cortisol en sangre, además de variables psicológicas mediante cuestionarios.

Para entender el dolor con una perspectiva más profunda analizaremos los datos pasando de una visión de variables independientes o correlaciones por pares de variables que afectan a un proceso, a un espacio multi-dimensional **donde la interacción entre las variables medidas y el patrón que forman lo que caracteriza el proceso que se estudia**.

Sin tomar en consideración la interacción de los componentes la visión del problema quedaría incompleta y distorsionada[5].

Se genera un espacio para la consideración de dependencias insospechadas entre variables (teoría de redes) y con ello se da la posibilidad matemática de explorar las diferencias sutiles entre grupos o condiciones (máquinas de aprendizaje). La representación gráfica y multi-dimensional del problema permite estudiar las reacciones en cadena que subyacen a la evolución de un sistema, y deja la puerta abierta a entender dinámicas caóticas donde una breve o leve diferencia puede resultar tras la evolución temporal o espacial

en grandes diferencias de procesos (teoría de caos, sistemas complejos, atractores y bifurcaciones). Pretendemos estudiar los mecanismos de un modelo biopsicosocial del dolor de espalda utilizando metodología matemática avanzada para poder diseñar tratamientos que sean más efectivos desde un punto de vista clínico, económico y social.

Gran parte del programa MBSR se enfoca en aprender a prestar atención a las sensaciones corporales mediante ejercicios de atención voluntaria en posición supina, sedente o semisupina, estiramientos suaves con un énfasis a centrar conscientemente la atención al cuerpo, junto con discusiones y prácticas orientadas a aplicar la atención plena a las experiencias de la vida diaria, incluyendo los estresores. El programa MBSR se usó inicialmente para tratar pacientes con dolor crónico (Kabat-Zinn 1982), pero desde entonces se ha aplicado a muchas otras poblaciones adultas de pacientes y comunidades (Ludwig & Kabat-Zinn 2008).

2.2 OBJETIVOS

Objetivo Principal

- Estudio de la influencia del estrés en la inflamación y la sintomatología del paciente con dolor de espalda.

Objetivos Secundarios

- Estudio de la influencia del entrenamiento mental en la modulación del dolor y el malestar en pacientes con discopatía.
- Desarrollo de un modelo psico-bio-social del dolor a partir de variables psicológicas, demográficas y biológicas utilizando algoritmos matemáticos de inteligencia artificial.
- Estudio de la influencia del estrés en la inflamación de la vía aérea superior y la influencia del entrenamiento mental en la mejoría del Síndrome de Apnea-Hipoapnea del sueño.
- Estudio de la influencia del entrenamiento mental en la reducción de la frecuencia respiratoria y su repercusión en la mejoría de la apnea.

3 TIPO DE ESTUDIO Y DISEÑO

3.1 Tipo de estudio.

Ensayo clínico randomizado.

3.2 Proceso de aleatorización

La asignación al azar de ambos tratamientos se realizará mediante una aleatorización por bloques de dos. El proceso de aleatorización se llevará a cabo utilizando un programa informático específico (Qualtrics) y se realizará de forma independiente para cada uno de los centros.

Una vez obtenido el consentimiento informado, a cada sujeto que cumpla con los criterios de inclusión y exclusión se le asignará un número de paciente.

Dado que el clínico que evaluará la respuesta será ciego a la asignación de los tratamientos, la correspondencia entre el tratamiento aplicado y el número de paciente permanecerá en un documento custodiado por el investigador principal en cada centro, y no será conocido de forma general hasta que haya concluido el tratamiento de los datos o se haya producido un acontecimiento adverso grave.

3.3 Tipo de control y diseño

Ensayo clínico multicéntrico, aleatorizado, ciego para evaluadores de la respuesta y controlado con el tratamiento usual según el diagnóstico de cada paciente.

3.4 Técnicas de enmascaramiento

El tipo de enmascaramiento empleado en el estudio será el abierto pero ciego para los evaluadores de la respuesta.

Con el objeto de poder mantener el ciego de los evaluadores, el cuaderno de recogida de datos será únicamente identificado mediante el número de paciente, no conteniendo ninguna información sobre el tratamiento recibido.

Las variables medidas serán trasladadas a una base de datos, anónima, en un formato estándar.

4 POBLACION DE ESTUDIO

Pacientes adultos diagnosticados con discopatía reciente (menos de 2 años del diagnóstico y sintomatología). Los participantes deben aportar un informe médico que acredite la discopatía.

Se incluirán pacientes con sintomatología de dolor y con diagnóstico:

1. Discopatía degenerativa en cualquier región de la columna
2. Hernia discal en cualquier región de la columna

5 PROCEDIMIENTO DE RECLUTAMIENTO DE PACIENTES

1.Captación:

a. Mediante mailing list a la lista de pacientes y personal del BTI
b. Mediante mailing list a la lista de clientes de nirakara institute.
c. Mediante mailing list y cartelería en la Universidad complutense de Madrid
d.

2.En las diferentes campañas de captación se indicarán claramente los objetivos de la investigación, los criterios de inclusión y el compromiso adquirido por parte del paciente.

3.Los sujetos interesados deberán rellenar un cuestionario online (mediante el software especializado Qualtrics) cuyas preguntas hacen referencia a los criterios de exclusión.

4.Todas las organizaciones involucradas tienen una cuenta en qualtrics y los cuestionarios y datos serán compartidos por todos en tiempo real.

5.Se analizan los datos recogidos según la idoneidad clínica de los pacientes, según los criterios de inclusión y exclusión.

6.A los sujetos que no cumplan con los criterios de exclusión e inclusiójn se les informa de la decisión y del tratamiento de los datos aportados.

7.A los sujetos que cumplen con los criterios de exclusión e inclusión se les informa vía telefónica y se les realiza una entrevista estructurada siguiendo el formato del cuestionario de inclusión/exclusión. Se indicará claramente al paciente que es libre de participar, y libre de abandonar el estudio en cualquier momento, si así lo desea, sin tener que justificar su decisión, y sin que ello suponga detrimento en su seguimiento clínico.

8.Se enviará en formato digital (Adobe Sign) los documentos del consentimiento informado. Dejando el tiempo necesario para leer y preguntar lo que estime oportuno. En el momento de la firma el software envía automáticamente una copia del documento firmado a todos los firmantes. Adobe cumple con todos los requerimientos de la nueva GDPR Europea.

9.Se incluye al paciente en el ensayo.

10.Los sujetos se codifican y se aleatorizan mediante un software online (Qualtrics). Todas las organizaciones involucradas tienen acceso online al proceso de aleatorización.

11.Se informa al paciente, mediante correo electrónico y llamada telefónica del procedimiento a seguir: Cuestionarios a cumplimentar, días (pre-post) de recogida de muestras, horario específico de la intervención para las personas del grupo experimental.

6 SELECCIÓN DE LOS SUJETOS

Serán reclutados pacientes adultos con discopatías, que cumplan los siguientes criterios de inclusión y exclusión:

6.1 CRITERIOS DE INCLUSIÓN

Los pacientes serán elegibles para su inclusión en este estudio si se cumplen los siguientes criterios:

1. Estar diagnosticado de discopatía sintomatológica (Los participantes deben aportar un informe médico que acredite la discopatía).

2. Movilidad normal ó moderada:

- ***Capacidad funcional normal:*** Capacidad funcional para desarrollar las acciones habituales sin limitación a pesar del dolor.

- ***Capacidad funcional reducida:*** Capacidad funcional restringida a algunas actividades cotidianas.

3. Estado cognitivo normal.

4. Pacientes que hayan leído y firmado previamente el consentimiento informado.

7. Pacientes capaces y estar dispuestos a cumplir con los procedimientos del estudio.

6.2 CRITERIOS DE EXCLUSIÓN

Los pacientes serán elegibles para su inclusión en este estudio si no se cumple ninguno de los siguientes criterios:

1. Psicopatologías graves

2. Pensamientos suicidas.

3. Depresión profunda.

4. Psicosis.

5. Drogadicción.

6. **Capacidad funcional muy limitada** y alteración cognitiva: Enfermos confinados en la cama o una silla. Necesitan la ayuda de una tercera persona.

7. No incorporar tratamientos clínicos, psicológicos o farmacológicos diferentes a los que utilizaban para el dolor, durante el programa, ni cambios drásticos en el estilo de vida.

6.3 Número de sujetos previstos

Dado un nivel de confianza del 95%, un margen de error del 5% y asumiendo una distribución normal centrada en un incremento de reducción del estrés del 20-25% en los participantes respecto al grupo control, se estima un tamaño muestral de 96 participantes (48 por grupo con medidas longitudinales). Para garantizar que el protocolo MBSR se hace adecuadamente es aconsejable un número no superior de 25 participantes por curso, por lo que la muestra experimental será dividida en dos grupos semanales de 24 personas.

6.4 Criterios de retirada y análisis de los mismos

Cada paciente podrá abandonar el estudio en cualquier momento, sin tener que exponer razones y sin sufrir ninguna desventaja personal (esta afirmación se recoge también explícitamente en la “Hoja de información al paciente”).

Serán **excluidos** del estudio aquellos pacientes potencialmente elegibles pero que no cumplan los criterios de inclusión o no hayan proporcionado previamente su consentimiento. Se considerarán como **retiradas**, a los

posibles sujetos incluidos en el estudio pero que no pueden ser analizados por haber sido incluidos por error, incumplimiento del tratamiento, desviaciones del protocolo, etc.

Se creará una Base de Datos informatizada con un sistema estandarizado de tratamiento de bases de datos con el fin de facilitar el acceso y comprobación en cualquier momento del proceso. Dicha base estará dotada con rangos y filtros para detectar y evitar inconsistencias y errores y donde las variables no registradas aparecerán codificadas como "missing" y serán tratadas matemáticamente antes de su análisis.

7 TRATAMIENTOS DEL ESTUDIO

7.1 Tratamientos del estudio

- **Tratamiento experimental**: Se utilizará Mindfulness Based Stress Reduction (MBSR), una intervención psicopedagógica validada para la reducción del estrés.

  Programa psico-educativo de reducción del estrés basado en mindfulness. Programa de 30 horas de duración repartidas en 9 sesiones con una frecuencia semanal de 2,5 h y una sesión intensiva entre la semana 6 y 7 del programa con una duración de 7,5 horas. Cada semana se cubren los contenidos teóricos necesarios para comprender las prácticas de desarrollo atencional propuestas en las intervenciones basadas en mindfulness. Además, se explican conceptos básicos de la psicobiología y psicología del estrés y el dolor. Los sujetos tendrán una carga de prácticas para casa de 45 minutos diarios aproximadamente durante el tiempo que dure la intervención.

- **Tratamiento control**: Los participantes asignados al grupo control seguirán el tratamiento usual, según su diagnóstico.

  Se pretende testar la diferencia entre el tratamiento experimental y los procedimientos estándar hasta ahora aplicados en la clínica. Dichos programas de rehabilitación física tienen por objeto reducir la sensación de dolor y como efecto colateral el estrés producido por el mismo.

A los pacientes del grupo control se les ofrecerá terapia de MBSR a la finalización del estudio.

7.2 Tratamientos no permitidos

Durante la duración del programa se pedirá a los participantes que no modifiquen sus hábitos clínicos ni inicien nuevos tratamientos farmacológicos o clínicos. En caso positivo el participante podrá seguir disfrutando del programa, pero no será incluido en el análisis, para evitar interferencias con las variables a ser testadas.

8 VARIABLES DE ESTUDIO

VARIABLE DE EXPOSICIÓN: Intervención psicoeducativa para la reducción del estrés en pacientes con dolor de espalda

8.1 VARIABLES DEMOGRÁFICAS:

- *Nº de paciente*
- *Género*
- *Edad*
- *Nacionalidad*
- *País de residencia*
- *Estado civil*
- *Nivel de estudios*
- *Situación laboral*
- *Práctica previa de mindfulness*

8.2 VARIABLES DE RESULTADO:

8.2.1 PRIMARIAS

- **Reducción del estrés**. Medido con la variación del cortisol en sangre.
- **Variación de Citoquinas** ((TNF)-α e interleukina (IL)-1β, IL-6 y IL17), como biomarcador aproximado de la inflamación. Marcadores bio-químicos de la discopatía: Citoquinas ((TNF)-α, interleukin (IL)-1β, IL-6 and IL-17), y medidas de cortisol. Dichas muestras se extraen de un análisis

de sangre que se realizará a los participantes antes y después de iniciar los programas tanto experimental como control. Dichas muestras serán analizadas por Laboratorios Echevarne.

- **Variación del índice de apneas nocturnas**.
  Análisis de sueño y respiración: La realización de las pruebas de registro del sueño y respiración se llevará a cabo en el hogar del paciente. El estudio del sueño se realizará con una poligrafía respiratoria validada (BTI-APNiA®). Este dispositivo tiene siete canales: flujo respiratorio, frecuencia respiratoria, saturación de oxígeno, frecuencia cardiaca, posición corporal, ronquidos y esfuerzo torácico-abdominal.
  El dispositivo mide el flujo de aire nasal y frecuencia respiratoria con una sonda conectada a un transductor y la saturación de oxígeno con oximetría de pulso cutánea mediante una sonda de dedo Todos los estudios del sueño serán analizados automáticamente por BTI-APNiA® según los criterios de la American Academy of Sleep Medicine. El análisis del sueño será controlado por un técnico del sueño y supervisado por un especialista en medicina del sueño.

8.2.2 SECUNDARIAS

- **Variables psicológicas mediante cuestionarios autoinformados:** mindfulness, dolor percibido, aceptación del dolor, catastrofización del dolor, afrontamiento de dolor, fatiga por dolor, discapacidad, impacto del dolor en el sueño, consciencia corporal, estilo de vida, satisfacción vital, estado de ánimo.

  - **MINDFULNESS Y COMPASIÓN:**

    - Five Facet Mindfulness Questionnaire (FFMQ): escala de 39 ítems que ofrece una puntuación total del nivel de mindfulness de una persona. Se divide a su vez en 5 subescalas: no-reactividad a la experiencia interna, observación, actuación con conciencia, descripción y no juicio. Los estudios de fiabilidad muestran una muy buena consistencia interna con valores de alfa de Cronbach que oscilan entre 0,85 y 0,92 (Baer et al. 2006).

    - Self-Compassion Scale (SCS): evalúa la capacidad de la persona para ser amable y comprensiva consigo misma ante los fallos en lugar de ser autocrítico, percibir la experiencia propia como parte de la experiencia humana en vez de aisladamente y ser consciente de los pensamiento y sentimientos dolorosos en lugar de sobre identificarse con

ellos. La escala está formada por 26 ítems puntuados en una escala tipo likert de 5 puntos. Los estudios de fiabilidad muestran una buena consistencia interna con valores de alfa de Cronbach en torno a 0,80 en todas las subescalas (Neff, 2003).

- **SINTOMATOLOGÍA PSICOLÓGICA:**

  - Perceived Stress Scale (PSS): medida de autoinforme diseñada para evaluar el nivel de estrés percibido durante el último mes. Consta de 14 ítems puntuados en una escala tipo likert de 0 al 4. Los estudios de fiabilidad muestran valores de alfa de Cronbach superiores a 0,70 en todas sus versiones (Cohen, Kamarck, & Mermelstein, 1983).

  - DASS-21: escala diseñada para evaluar el estado de ánimo negativo mediante tres subescalas: estrés, ansiedad y depresión. La escala cuenta con 21 ítems puntuados en una escala tipo Likert de 0 a 3. Los estudios de fiabilidad muestran una muy buena consistencia interna con valores de alfa de Cronbach de 0,93 (Henry, & Crawford, 2005).

- **EVALUACIÓN DEL DOLOR:**

  - Chronic Pain Grade Scale (CPGS; 90): es una medida multidimensional diseñada para evaluar dos dimensiones de la severidad del dolor crónico: la intensidad del dolor y la discapacidad provocada por el dolor. Es una medida muy utilizada para la evaluación del dolor crónico musculo esquelético y dolor de espalda. La escala está formada por 7 ítems puntuados en una escala Likert de 0 a 10. Los estudios de fiabilidad han mostrado valores de alfa de Crombach de 0,74 (Von Korff, Ormel, Keefe & Dworkin, 1992).

  - Brief Pain Inventory (BPI): medida diseñada para medir tanto la intensidad del dolor (dimensión sensorial) como la interferencia del dolor en la vida del paciente (dimensión reactiva) en pacientes con dolor crónico provocado por osteroartritis, dolor de espalda y cáncer. También evalúa la localización del dolor, la medicación empleada para el dolor y el alivio del dolor en las últimas 24 horas. Los estudios de fiabilidad muestran valores de alfa de Cronbach que oscilan entre 0,77 y 0,91 (Jensen, Thornby, & Shanti, 2004).

  - Chronic Pain Acceptance Questionnaire-Revised (CPAQ-R): instrumento diseñado para evaluar la aceptación del dolor. La aceptación del dolor es un constructo opuesto a los

intentos de evitar o tratar de controlar el dolor que generan una hiperfocalización y una maximización de las sensaciones de dolor. Esta escala consta a su vez de dos subescalas: compromiso con las actividades (búsqueda de actividades importantes para tu vida a pesar del dolor) y disposición del dolor (reconocimiento de que la evitación y los intentos de control a menudo son infructuosos e incrementan el dolor). La escala está formada por 20 ítems puntuados en una escala tipo likert de 0 a 6. Los estudios de fiabilidad muestran una buena consistencia interna con valores de alfa de Cronbach de 0,82 para la escala de Compromisos con las Actividades y de 0,78 para la escala de Predisposición ante el Dolor (McCraken, Vowles, & Eccleston, 2004).

- Pain Catastrophising Scale (PCS): evalúa la tendencia a magnificar la amenaza de un estímulo doloroso y el sentimiento de impotencia en presencia del dolor, así como la incapacidad para prevenir o inhibir pensamientos relacionados con el dolor (tanto antes, como durante, como después del evento doloroso). Este instrumento se divide a su vez en tres subescalas: rumiación del dolor, magnificación del dolor y sentimiento de desesperanza a la hora de manejar el dolor. La escala está formada por 13 ítems puntuados en una escala tipo likert de 0 a 4. Los estudios de fiabilidad muestran una buena consistencia interna con valores de alfa de Cronbach que oscilan entre 0,87 y 0,93 (Sullivan Bishop, & Pivik, 1995).

- Brief Fatigue Inventory (BFI): el objetivo de esta escala es evaluar la severidad de la fatiga y el impacto de la fatiga producida por el dolor en el funcionamiento cotidiano de las personas. La escala está formada por 9 ítems puntuados en una escala tipo likert de 0 a 10. Los estudios de fiabilidad muestran una muy buena consistencia interna con valores de alfa de Cronbach que oscilan entre 0,82 y 0,97 (Mendoza et al., 1999).

- Pain Self–Efficacy Questionnaire (PSEQ): evalúa en qué medida una persona con dolor cree que es capaz de realizar sus actividades diarias independientemente de su dolor. La escala está formada por 10 ítems puntuados en una escala tipo likert de 0 a 6. Los estudios de fiabilidad muestran una muy buena consistencia interna con un valor de alfa de Cronbach de 0,92 (Nicholas, 1989; Nicholas, 2007).

- Survey of Pain Attitudes - Brief (SOPA-B): evalúa la actitud del paciente ante siete dimensiones de la experiencia del dolor crónico: control del dolor, discapacidad asociada al dolor, claves médicas del dolor, solicitud de la ayuda de otros ante el dolor, medicación para

el dolor, la influencia de las emociones en el dolor y el dolor como indicador del daño físico. La escala está formada por 30 ítems puntuados en una escala tipo likert de 0 a 4. Los estudios de fiabilidad muestran una buena consistencia interna con valores que oscilan entre 0,70 y 0,83 para las diferentes subescalas (Jensen et al., 1987; Tait, & Chibnall, 1997).

- Pain Coping Inventory (PC): instrumento diseñado para evaluar las estrategias de afrontamiento ante el dolor que emplean los pacientes con dolor crónico. El inventario incluye seis subescalas que evalúan los siguientes componentes: transformación del dolor, distracción, reducción de las demandas, retirada, preocupación y descanso. Estos componentes a su vez pueden agruparse en dos dimensiones: afrontamiento activo y pasivo ante el dolor. La escala está formada por 34 ítems puntuados en una escala tipo likert de 1 a 4. Los estudios de fiabilidad muestran una buena consistencia interna con valores superiores a 0,70 para las diferentes subescalas (Kraaimaat, & Evers, 2003).

➢ **FUNCIONAMIENTO / DISCAPACIDAD:**

- Short Form-36 Bodily Pain Scale (SF-36 BPS): es una de las ocho subescalas del cuestionario SF-36 (Ware, Kosinski & Keller, 1994) diseñado como una medida genérica del estado de salud para estudios poblacionales. La subescala SF-36 BPS está constituida por dos ítems que miden la interferencia del dolor en las actividades de la vida diaria (McHorney, Ware & Raczek, 1993). Los estudios de fiabilidad muestran valores de consistencia interna mediante alfa de Cronbach que oscilan entre 0,72 y 0,77 (Quintana et al., 2005; Escobar et al., 2007).

- Pain and Sleep Questionnaire (PSQ): evalúa el impacto del dolor crónico en la calidad del sueño. La escala está formada por 8 ítems puntuados en una escala visual analógica del 0 al 100. Los estudios de fiabilidad muestran una consistencia interna adecuada (Peloso et al., 2000; Watson, 1998).

- **BIENESTAR:**

  - Satisfaction with Life questionnaire (SWLS): escala breve de 5 ítems diseñada para medir los juicios cognitivos globales de una persona sobre su satisfacción con la vida. Los estudios de fiabilidad muestran una buena consistencia interna con valores de alfa de Cronbach de 0,78 (Diener, Emmons, Larsen, & Griffin, 1985).

  - WHO-5 Well Being Index: escala de 5 ítems diseñada para evaluar el bienestar psicológico subjetivo de la persona. Los estudios de fiabilidad muestran una consistencia interna adecuada (Bech, 2004; Topp et al., 2015).

8.3 COVARIABLES

1. Medidas de implicación en el programa psicoeducativo: práctica diaria y asistencia.

REFERENCIAS de los cuestionarios

Von Korff M, Ormel J, Keefe FJ, Dworkin SF. Grading the severity of chronic pain. Pain 1992;50:133–49.

Ware JE, Kosinski M, Keller SK. SF-36 physical and mental health summary scales: a user's manual. Boston: The Health Institute; 1994.

McHorney CA, Ware JE, Raczek AE. The MOS 36-Item Short-Form Health Survey (SF-36) II: psychometric and clinical tests of validity in measuring physical and mental health constructs. Med Care 1993;31: 247–63.

Quintana JM, Escobar A, Bilbao A, Arostegui I, Lafuente I, Vidaurreta I. Responsiveness and clinically important differences for the WOMAC and SF-36 after hip joint replacement. Osteoarthritis Cartilage 2005;13:1076–83.

Escobar A, Quintana JM, Bilbao A, Arostegui I, Lafuente I, Vidaurreta I. Responsiveness and clinically important differences for the WOMAC and SF-36 after total knee replacement. Osteoarthritis Cartilage 2007;15:273–80.

Tan, G., Jensen, M. P., Thornby, J. I., & Shanti, B. F. (2004). Validation of the Brief Pain Inventory for chronic nonmalignant pain. The Journal of Pain, 5(2), 133-137.)

Cohen, S., Kamarck, T., & Mermelstein, R. (1983). A global measure of perceived stress. *Journal of Health and Social Behavior, 24*, 385-396.

Henry, J. D., & Crawford, J. R. (2005). The short-form version of the Depression Anxiety Stress Scales (DASS-21): Construct validity and normative data in a large non-clinical sample. British journal of clinical psychology, 44(2), 227-239.

McCraken, L. M., Vowles, K. E. & Eccleston, C. (2004). Acceptance of chronic pain: component analysis and a revised assessment method. Pain, 107, 159-166

Sullivan MJL, Bishop SR, Pivik J. The Pain Catastrophizing Scale: Development and Validation. Psychol Assess, 1995; 7(4): 524-32.

Mendoza TR, Wang XS, Cleeland CS, Morrissey M, Johnson BA, Wendt JK, et al. The rapid assessment of fatigue severity in cancer patients: Use of the Brief Fatigue Inventory. Cancer. 1999;85(5):1186–96.

Nicholas MK. The pain self-efficacy questionnaire: Taking pain into account. Eur J Pain. 2007;11:153–63.

Nicholas, M. K. (2007). The pain self-efficacy questionnaire: Taking pain into account. European journal of pain, 11(2), 153-163.

Jensen, M.P., Karoly, P. and Huger, R., The development and preliminary validation of an instrument to assess patients' attitudes toward pain, J. Psychosom. Res., 31 (1987) 393–400.

Tait, R. C., & Chibnall, J. T. (1997). Development of a brief version of the Survey of Pain Attitudes. Pain, 70(2-3), 229-235.

Kraaimaat, F. W., & Evers, A. W. (2003). Pain-coping strategies in chronic pain patients: psychometric characteristics of the pain-coping inventory (PCI). International journal of behavioral medicine, 10(4), 343-363.

Peloso P, Bellamy N, Bensen W, et al. Double blind randomised placebo control trial of controlled release codeine in the treatment of osteoarthiritis of the hip or knee. J Rheumatol 2000;27:764-71.

Watson CP BN. Efficacy of oxycodone in neuropathic pain: A randomized trial in postherpectic neuralgia. Neurology 1998;50:1837-41.

Diener, E., Emmons, R. A., Larsen, R. J., & Griffin, S. (1985). The Satisfaction with Life Scale. Journal of Personality Assessment, 49, 71-75.

Bech, P. (2004). Measuring the dimension of psychological general well-being by the WHO-5. Quality of Life Newsletter, 15-16.

Topp, C. W., Østergaard, S. D., Søndergaard, S., & Bech, P. (2015). The WHO-5 Well-Being Index: a systematic review of the literature. Psychotherapy and psychosomatics, 84(3), 167-176.

Baer, R. A., Smith, G. T., Hopkins, J., Krietemeyer, J., & Toney, L. (2006). Using self-report assessment methods to explore facts of mindfulness. Assessment, 13, 27–45. doi:10.1177/1073191105283504.

Neff, K. D. (2003). The development and validation of a scale to measure selfcompassion. Self and Identity, 2(3), 223–250.

8.4 Evaluación de la respuesta

La base de datos de cada participante consta de 3 bloques de medidas: Demográficas, psicológicas, y biológicas.

1.- Pre-procesamiento: Los valores "missing", es decir aquellos que no han sido aportados por el participante o en el caso de las medidas biológicas, que no hayan podido ser medidos, se estimaran mediante un algoritmo multivariable no lineal de imputación de datos. Solo serán imputados los valores de las medias "post", aquellas tomadas después de haber realizado el programa.

2.-Relaciones de dependencia entre variables. Se estima la correlación lineal entre cada par de variables para el grupo en las condiciones Pre, antes de realizar el programa MBI o control, y Post, después de concluir dichos programas. El coeficiente de correlación se estima usando el algoritmo Kendall, menos sensible a outliers estadísticos que el coeficiente de correlación Pearson. Se tomará como correlación aquella que cumpla los criterios estadísticos del 0.01 sobre el valor p. De esta forma tenemos una matriz semi-binaria donde el elemento (i,j) de la matriz será 0 en caso de correlación no significativa o el correspondiente valor de correlación entre las variables de posiciones i y j. La matriz será simétrica y con diagonal nula.

3.-Las matrices de relación entre variables serán consideradas redes o grafos, permitiendo así la estimación de parámetros topológicos como la determinación de qué variables tienen un mayor papel intermediario, mayor coeficiente de agrupamiento o mayor capacidad de difusión de información en la red, así como parámetros globales como la eficiencia de la red. Se consideran 2 redes: Red psicológica, red demográfica y red biológica, para posteriormente considerar una red multi-capa compuesta por las tres redes anteriores y sus relaciones. Como estadístico topológico se normalizarán las medidas a aquellas generadas por una red aleatoria que cumpla los criterios del mismo número de nodos y conexiones, pero con arquitecturas generadas por el método de barajeado mil veces, con el objetivo de establecer un umbral de significación estadística.

4.- Utilizando máquinas de aprendizaje de inteligencia artificial se evaluará la capacidad predictiva de dicha red, con el objetivo de extraer biomarcadores del modelo psico-bio-social de la discopatía y comprobar la eficacia de los programas de reducción del estrés basado en mindfulness. En ese paso se comparan los cambios inducidos por los grupos experimentales y control, para la validación de las variables hipotetizadas como mediadoras del bienestar.

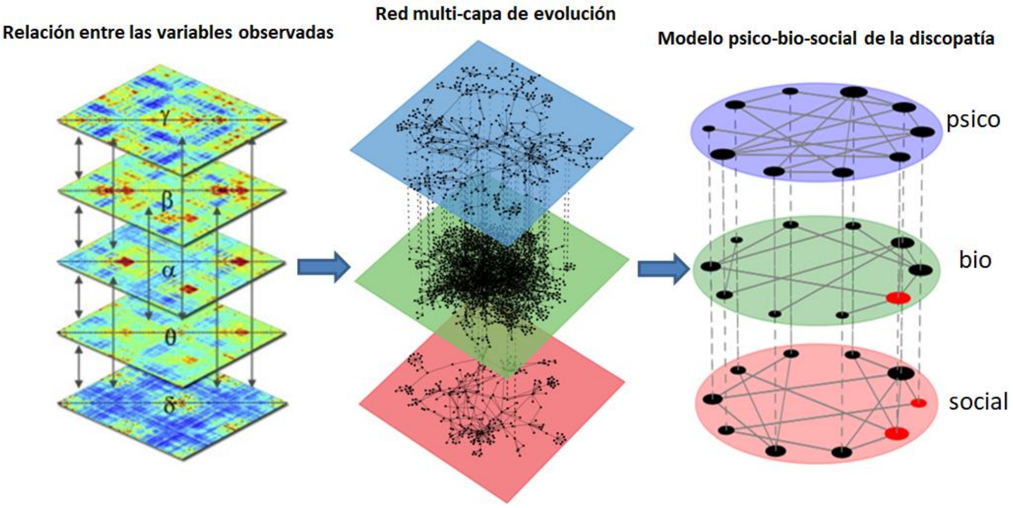

Figura: La relación entre todas las variables medidas, psicológicas, demográficas-sociales y biológicas nos proporciona un conjunto de matrices con los coeficientes de correlación entre ellas (panel de la izquierda). Dicha información es tratada como 3 redes de diferente naturaleza que conectan entre ellas (panel central) para posteriormente (panel derecho) extraer qué variables han sido determinantes en la evolución de los participantes en el programa de reducción del estrés basados en mindfulne

9 DESARROLLO DEL ESTUDIO-CRONOGRAMA

	Visita reclutamiento	Visita diagnóstica	Visita inclusión	Visitas TTo 1 -9	Visitas Seguimiento (3-6 meses post)
Información al paciente	X				
Consentimiento informado		X			
Historia clínica		X			
Analítica sanguínea		X			X
Criterios de inclusión			X		
Reclutamiento (nº de paciente)			X		
Asignación del grupo de tratamiento (aleatorización)			X		
Análisis de sueño y respiración			X		X (6 meses)
Fase de Tratamiento				X	X
Evaluación de eficacia				X	X
Evaluación seguridad				X	X

9.1 Visita reclutamiento

En esta visita se informará al paciente respecto a la realización del ensayo clínico, así como de las características de este. En el caso de que el paciente se muestre interesado en participar en el mismo se le dará una hoja de información al paciente y un formulario de consentimiento informado.

9.2 Visita diagnóstica

En esta visita se procederá a recoger el consentimiento informado firmado de los pacientes que hayan decidido participar en el estudio.

Se procederá a solicitar una analítica.

Se pedirá a los pacientes un informe médico que acredite la discopatía.

9.3 Visita inclusión

En esta visita se procederá a comprobar el grado de cumplimiento de los criterios de inclusión/exclusión partiendo de los datos recogidos en la historia clínica y en la analítica realizada. Aquellos pacientes que cumplan con dichos criterios serán incluidos en el estudio y se les asignará un número de paciente en el estudio, de acuerdo con el listado de aleatorización.

Se dedicarán 15 minutos para entregar el equipo APNIA y explicar su funcionamiento.

Se citará al paciente para la próxima visita.

9.4 Visita tratamiento

Programa MBSR

Programa de 30 horas de duración repartidas en 9 sesiones con una frecuencia semanal de 2,5 h y una sesión intensiva entre la semana 6 y 7 del programa con una duración de 7,5 horas. Cada semana se cubren los contenidos teóricos necesarios para comprender las prácticas de desarrollo atencional propuestas en las

intervenciones basadas en mindfulness. Además, se explican conceptos básicos de la psicobiología y psicología del estrés y el dolor. Los sujetos tendrán una carga de prácticas para casa de 45 minutos diarios aproximadamente durante el tiempo que dure la intervención.

Se recordará a los pacientes traer el equipo APNIA antes de la primera sesión de MBSR.

9.5 Visita seguimiento

Para testar los efectos duraderos del programa se medirán las variables de estudio 3 y 6 meses después de haber finalizado el programa.

9.6 Finalización del ensayo clínico

La finalización del ensayo clínico será cuando finalice la última visita del último sujeto reclutado. (siglas LVLS en inglés).

10 SEGURIDAD

Todas las sospechas de reacciones adversas graves e inesperadas (RAGI) se notificarán de acuerdo con la normativa vigente sobre ensayos clínicos en España, a la AEMPS a los CEIC, a las autoridades sanitarias de las CCAA y a los investigadores, en los plazos y forma establecidos: inferior a 15 días o a 7 días si se ha producido la muerte.

Se reservará un espacio del CRD para la anotación de cualquier circunstancia que pudiera ser considerada como acontecimiento adverso, así como sus características (inicio, fin, etc.).

11 SISTEMA DE ALMACENAMIENTO Y CONTROL DE ACCESO A LA INFORMACIÓN

Los datos relacionados con las muestras biológicas de origen humano serán almacenados con fines de investigación biomédica en una base de datos que custodiará el investigador principal de cada centro ya que es el único investigador que tendrá la correspondencia entre el código del paciente y sus datos identificativos. El paciente tendrá derecho a acceder a estos datos siempre que lo solicite.
En el caso de que el paciente lo solicite, el Investigador principal del centro donde se realiza el estudio, podrá proporcionarle información sobre los resultados de la investigación, una vez ésta haya finalizado.

12 DESTINO DE LA MUESTRA DE SANGRE TRAS SU UTILIZACIÓN EN ESTE ENSAYO

Una vez finalizado el estudio y según lo previsto en el **RD 1716/2011, de 18 de noviembre, sobre requisitos básicos de autorización y funcionamiento de los biobancos con fines de investigación biomédica**, las citadas muestras serán destruidas.

13 CONSIDERACIONES ÉTICAS

13.1 Consideraciones generales

El estudio se llevará a cabo de acuerdo con las normativas internacionales sobre estudios clínicos:

- La declaración de Helsinki en su última versión revisada [Fortaleza, 2013].
- Normas de Buena Práctica Clínica (ICH).
- Real Decreto 1090/2015 por el que se regulan los ensayos clínicos con medicamentos en España.
- Ley 14/2007 de Investigación Biomédica
- RD 1716/2011

El estudio sólo comenzará tras haber obtenido por escrito la autorización del Comité de Ética de la investigación con medicamentos de Euskadi antes del comienzo del mismo.

13.2 Información al paciente y consentimiento informado

Todos los voluntarios serán informados de las características del estudio verbalmente y por escrito mediante la HOJA DE INFORMACIÓN y que incluirá información sobre los siguientes aspectos del estudio clínico:

- Objetivo
- Metodología empleada
- Tratamiento administrado
- Beneficios esperados
- Incomodidades y riesgos derivados del estudio
- Posibles acontecimientos adversos

- Carácter voluntario de la participación y que pueden retirarse
- Acceso a los datos y confidencialidad
- Compensación económica
- Investigador responsable del estudio

Finalmente, tras ser ampliamente informado de las implicaciones y restricciones del protocolo, se solicitará a los sujetos que otorguen por escrito un consentimiento informado, junto al investigador principal, antes de iniciar el estudio.

Al firmar y fechar el formulario de consentimiento, el paciente declara su participación voluntaria y su intención de cumplir el Protocolo del Estudio y las instrucciones del Investigador y responder a las cuestiones que se planteen a lo largo del Estudio.

Adjuntos al presente protocolo se adjuntan los modelos de la hoja de información al voluntario y de la hoja de consentimiento informado.

El sujeto conservará a lo largo del estudio la hoja de información, con toda la información relevante del estudio, incluido el contacto con el investigador. El investigador conservará el consentimiento informado en el archivo del estudio.

13.3 Confidencialidad de los datos

Toda la información relativa a los datos de los pacientes incluidos en el estudio será estrictamente confidencial conforme con la Ley Orgánica de Protección de Datos (Ley 15/1999).

El investigador asegurará el mantenimiento del anonimato de los pacientes para lo que se procederá a registrar la identidad del paciente mediante un código alfanumérico de identificación en toda la documentación del estudio.

Podrán tener acceso a los datos confidenciales de los sujetos del estudio el equipo investigador y los representantes de las autoridades sanitarias competentes. El acceso a la base de datos informatizada con la información de las variables objeto del estudio también está permitido al estadístico que realice el análisis, por supuesto manteniendo el anonimato de los participantes.

Las historias clínicas de los sujetos participantes en el estudio serán custodiadas por el investigador en un lugar con llave.

Las copias del protocolo, cuadernos de recogida de datos, consentimientos informados y otros documentos pertenecientes al estudio deberán ser conservados en el archivo del estudio por el investigador durante el periodo de tiempo establecido por la legislación, reflejando el traspaso de datos en el momento que proceda.

Asimismo, y con el objeto de garantizar los derechos del paciente en cuanto a la confidencialidad de sus datos personales, los resultados del estudio siempre se presentarán de forma global y nunca, bajo ningún concepto, de forma individualizada.

13.4 Presupuesto del estudio

Los aspectos económicos del estudio se detallan en un anexo independiente y que supone un documento aparte.

13.5 Seguro

Dadas las características del estudio, no se considera necesario la contratación de un seguro. El centro clínico se responsabiliza de cubrir todos los gastos derivados de cualquier acontecimiento adverso debido a los tratamientos y/o las medidas de evaluación descritas en este protocolo.

14 CONSIDERACIONES PRÁCTICAS

14.1 Normas para el personal auxiliar que interviene en el estudio

El personal auxiliar que colabora en el estudio cumplirá las normas generales establecidas para la realización del estudio y seguirá en todo momento las instrucciones del investigador.

14.2 Manejo, correcciones y archivo de datos

Todos los datos relevantes de los sujetos del estudio se transcribirán con bolígrafo al Cuaderno de Recogida de Datos (CRD). Los CRD deben ser rellenados de forma completa y legible para facilitar el posterior procesamiento estadístico. Una vez finalizado el estudio, se evaluarán todos los datos recogidos en los CRD para la redacción del informe final.

En caso de ser necesaria alguna corrección, se tachará el dato incorrecto con una línea simple que permita su lectura y a su lado se escribirá el dato correcto, junto con la firma de quién realiza la corrección, sus iniciales y la fecha. Estas instrucciones aparecen en el CRD.

Toda la documentación referente al estudio permanecerá almacenada bajo custodia del Investigador Principal hasta la finalización del mismo, ocupándose asimismo de conservar los códigos de identificación de los pacientes durante el tiempo exigido por la reglamentación vigente. Asimismo, el promotor conservará toda documentación relativa al estudio durante el tiempo legalmente requerido.

Todos los datos y documentos se pondrán a disposición de las autoridades competentes si éstas así lo solicitan.

Se asegurará, en todo caso, la confidencialidad de los datos y documentos contenidos en el archivo.

14.3 Condiciones de publicación

El equipo investigador podrá presentar los resultados en una reunión científica apropiada y/o publicarlos en una revista de reconocido prestigio, comprometiéndose a solicitar con carácter previo la conformidad del promotor mediante carta certificada; en su solicitud de conformidad, el investigador deberá adjuntar una copia del manuscrito u original, a efectos de que el monitor pueda conocer dicha información o material informativo.

El promotor deberá responder por escrito en un plazo máximo de treinta días, comunicando su autorización, sus reservas o disconformidad sobre la publicación de la referida información. Transcurrido dicho plazo sin obtener respuesta se entenderá que el silencio es la tácita autorización para su difusión.

Si el Promotor así lo solicita, con el fin de asegurar apropiadamente la protección de invenciones o desarrollos derivados del estudio, el Investigador Principal acepta retrasar la presentación de la publicación propuesta, durante un plazo no superior a 6 meses

Los investigadores podrán incluir el título del estudio en su respectivo *Curriculum Vitae*.

15 BIBLIOGRAFÍA

Cherkin DC, Sherman KJ, Balderson BH, Cook AJ, Anderson ML, et al. 2016. Effect of Mindfulness-Based Stress Reduction vs Cognitive Behavioral Therapy or Usual Care on Back Pain and Functional Limitations in Adults with Chronic Low Back Pain: A Randomized Clinical Trial. *JAMA*. 315(12):1240–49

Kabat-Zinn J. 1982. An outpatient program in behavioral medicine for chronic pain patients based on the practice of mindfulness meditation: Theoretical considerations and preliminary results. *Gen. Hosp. Psychiatry*. 4(1):33–47

Ludwig DS, Kabat-Zinn J. 2008. Mindfulness in medicine. *J. Am. Med. Assoc.* 300(11):1350–52. Annotated Reference: an early and important review of the mindfulness intervention health literature.

Morone NE, Greco CM, Moore CG, Rollman BL, Lane B, et al. 2016. A mind-body program for older adults with chronic low back pain: A randomized clinical trial. *JAMA Intern. Med.* 176(3):329–37

Schwartz L, Slater MA, Birchler GR. 1994. Interpersonal stress and pain behaviors in patients with chronic pain. *J. Consult. Clin. Psychol.* 62(4):861–64
