# Supplementary material for: The effect of mindfulness on the inflammatory, psychological and biomechanical domains of adult patients with low back pain: A randomized controlled clinical trial
Source: PLoS One. 2022 Nov 9;17(11):e0276734. doi: 10.1371/journal.pone.0276734 (PMC9645607; doi:10.1371/journal.pone.0276734)
Supplement: S4 File — (PDF) [file pone.0276734.s004.pdf]

3 TYPE OF STUDY AND DESIGN

3.1 Type of study.

Randomised clinical trial.

3.2 Randomisation process

The random assignment of both treatments will take place though randomisation in blocks of two. The randomisation process will be carried out using a specific computer programme (Qualtrics) and will be performed independently for each of the centres.

Once informed consent has been obtained, each subject who meets the inclusion and exclusion criteria will be assigned a patient number.

Given that the clinician who will evaluate the response will be blinded to the allocation of the treatments, correspondence between the treatment applied and the patient number will remain in a document kept by the head investigator at each centre, and will not be disclosed publicly until data processing is complete or in the case of a serious adverse event.

3.3 Type of control and design

Multicentre, randomised clinical trial with evaluators of the response blinded and controlled with the usual treatment according to each patient's diagnosis.

3.4 Masking techniques

The type of masking used in the study will be open but evaluators of the response will be blinded.

In an aim to keep the evaluators blinded, the data collection sheet will only include the patient number, not containing any information about the treatment received.

The variables measured will be transferred to a database, anonymised, in standard format.

4 STUDY POPULATION

Adult patients recently diagnosed with disc disease (less than 2 years since diagnosis and symptoms). Participants must provide a medical report stating that they have disc disease.

Patients with pain symptoms and the following diagnosis will be included:

1. Degenerative disc disease in any part of the spine
2. Herniated disc in any part of the spine

5 PATIENT RECRUITMENT PROCEDURE

1. Recruitment:
   a. Via a mailing list sent to BTI staff and patients list
   b. Via a mailing list sent to the Nirakara Institute client list.
   c. Via a mailing list and posters at the Complutense University of Madrid
   d.

2. In the different recruitment campaigns, the objectives of the research, the inclusion criteria and the commitment made by the patient will be clearly indicated.

3. Interested subjects fill in an online questionnaire (using the specialised Qualtrics software) featuring questions related to the exclusion criteria.

4. All of the organisations involved have a Qualtrics account and the questionnaires and data will be shared by everyone in real time.

5. The data collected is analysed according to the clinical suitability of the patients, with regard to the inclusion and exclusion criteria.

6. Subjects who do not meet the exclusion and inclusion criteria are informed of the decision and the processing of the data provided.

7. Subjects who meet the exclusion and inclusion criteria are informed by telephone and a structured interview is carried out with the same format as the inclusion/exclusion questionnaire. The patient will be clearly informed that they are free to participate, and free to leave the study at any time, if they wish to do so, without having to justify their decision and without detriment to their clinical monitoring.

8. The informed consent documents will be sent in digital format (Adobe Sign). Leaving enough time to read through them and ask any possible questions. When signed, the software automatically sends a copy of the signed document to all those who sign it. Adobe complies with all requirements of the new European GDPR.

9. The patient is included in the trial.

10. Subjects are coded and randomised using online software (Qualtrics). All organisations involved have online access to the randomisation process.

11. The patient is informed, by email and telephone call, of the procedure: Questionnaires to be filled in, days (pre-post) of sample collection, specific time of the intervention for the people in the experimental group.

6 SELECTION OF SUBJECTS

Adult patients with disc disease, who meet the following inclusion and exclusion criteria, will be recruited:

6.1 INCLUSION CRITERIA

Patients will be eligible to take part in this study if they meet the following criteria:

1. Have a symptomatic disc disease diagnosis (Participants must provide a medical report stating disc disease).

2. Normal or moderate mobility:

- ***Normal functional capacity:*** Functional ability to carry out habitual actions without limitation despite pain.

- ***Reduced functional capacity:*** Functional capacity restricted to some daily activities.

3. Normal cognitive state.

4. Patients who have previously read and signed the informed consent form.

7. Patients capable and willing to comply with the study procedures.

6.2 EXCLUSION CRITERIA

Patients will be eligible for inclusion in this study if they do not meet any of the following criteria:

1. Serious psychopathologies

2. Suicidal thoughts.

3. Severe depression.

4. Psychosis.

5. Drug addiction.

6. **Very limited functional capacity** and impaired cognitive functions: Patients confined to their beds or a chair. Those reliant on help from a third party.

7. Do not initiate clinical, psychological or pharmacological treatments other than those used for pain, during the programme, or make drastic changes in lifestyle.

6.3 Expected number of subjects

Given a confidence level of 95%, a margin of error of 5% and assuming a normal distribution centred on an increase in stress reduction of 20-25% in the participants compared to the control group, a sample size of 96 participants is estimated. (48 per group with longitudinal measurements). To guarantee that the MBSR protocol is followed properly, a number of no more than 25 participants per course is advisable, subsequently, the experimental sample will be divided into two weekly groups of 24 people.

6.4 Withdrawal criteria and subsequent analysis

Each patient is free to withdraw from the study at any time without needing to give an explanation and without suffering any personal disadvantages (this is also clearly stated in the "Patient Information Sheet").

Potentially eligible patients who do not meet the inclusion criteria or have not previously provided their consent will be **excluded** from the study.

Possible subjects included in the study but who cannot be analysed due to having been included by mistake, or due to non-compliance with treatment, deviations in protocol, etc., will be considered as **withdrawals**.

A Computerised Database will be created with a standardised database processing system to facilitate access and verification at any time during the process. This database will be equipped with ranges and filters to detect and avoid inconsistencies and errors and where unregistered variables will appear coded as "missing" and will be processed mathematically prior to their analysis.

7 STUDY TREATMENTS

7.1 Study treatments

- **Experimental treatment**: We will use Mindfulness Based Stress Reduction (MBSR), a validated psychopedagogical intervention for stress reduction.

  Psycho-educational stress reduction programme based on mindfulness. A 30-hour programme divided into 9 sessions, 2.5 hours every week, and an intensive session between week 6 and 7 of the programme that lasts for 7.5 hours. Each week, the theoretical content required to understand the attentional development practices proposed in the mindfulness-based interventions are covered. Also, basic concepts of the psychobiology and the psychology of stress and pain are explained. Subjects will have some exercises to do at home, approximately 45 minutes every day, for the duration of the intervention.

- **Control treatment**: Participants assigned to the control group will continue with their usual treatment, according to their diagnosis**.**

  The aim is to test the difference between the experimental treatment and the standard procedures applied so far in the clinic. These physical rehabilitation programmes are intended to reduce the feeling of pain and, as a collateral effect, the stress it produces.

The control group patients will be offered MBSR therapy upon completion of the study.

7.2 Treatments not permitted

For the duration of the programme, participants will be asked not to change their clinical habits or initiate new pharmacological or clinical treatments. If they do, the participant may continue to take part in the programme, but will not be included in the analysis, to avoid interference with the variables being tested.

8 STUDY VARIABLES

EXPOSURE VARIABLE: Psycho-educational intervention for stress reduction in patients with back pain

8.1 DEMOGRAPHIC VARIABLES:

- *Patient no.*
- *Gender*
- *Age*
- *Nationality*
- *Country of residence*
- *Marital status*
- *Education*
- *Employment status*
- *Previous experience with mindfulness*

8.2 RESULT VARIABLES:

8.2.1 PRIMARY

- **Reduction in stress**. Measured by the variation of cortisol in the blood.
- **Cytokine Variation** ((TNF)-α and interleukin (IL)-1β, IL-6 and IL17), as an approximate inflammation biomarker. Biochemical markers of disc disease: Cytokines ((TNF)-α, interleukin (IL)-1β, IL-6 and IL-17), and cortisol levels.

The samples are taken from a blood test that participants will undergo before and after starting the experimental and control programmes. The samples will be analysed by Laboratorios Echevarne.

- **Night apnoea index variation**.
  Sleep and breathing analysis: The sleep and breathing registration tests will be carried out at the patient's home. The sleep study will be performed with a validated respiratory polygraph (BTI-APNiA®). This device has seven channels: respiratory flow, respiratory rate, oxygen saturation, heart rate, body position, snoring and thoracic-abdominal effort.
  The device measures nasal air flow and respiratory rate with a probe connected to a transducer and oxygen saturation with skin pulse oximetry using a finger probe. All sleep studies will be automatically analysed by BTI-APNiA® in accordance with the criteria of the American Academy of Sleep Medicine. The sleep analysis will be controlled by a sleep technician and supervised by a sleep medicine specialist.

8.2.2 SECONDARY

- **Psychological variables using self-reported questionnaires:** mindfulness, perceived pain, pain acceptance, pain catastrophisation, capacity for coping with pain, pain-induced fatigue, disability, impact of pain on sleep, body awareness, lifestyle, life satisfaction, mood.

➢ **MINDFULNESS AND COMPASSION:**

- Five Facet Mindfulness Questionnaire (FFMQ): scale with 39 items offering a total score of a person's level of mindfulness. It is divided into 5 subscales: non-reactivity to inner experience, observation, acting with awareness, description and non-judgment. Reliability studies show very good internal consistency with Cronbach's alpha values ranging between 0.85 and 0.92 (Baer et al. 2006).

- Self-Compassion Scale (SCS): assesses the person's ability to be kind and understanding with themselves in the face of failure rather than being self-critical, to perceive one's own experience as part of the human experience rather than detached, and to be aware of painful thoughts and feelings rather than over-identifying with them.

The scale is made up of 26 items scored on a 5-point Likert-type scale. Reliability studies show good internal consistency with Cronbach's alpha values, around 0.80 in all subscales (Neff, 2003).

➢ **PSYCHOLOGICAL SYMPTOMS:**

- Perceived Stress Scale (PSS): self-report measure designed to assess the level of perceived stress during the past month. It consists of 14 items scored on a Likert-type scale from 0 to 4. Reliability studies show Cronbach's alpha values greater than 0.70 in all versions (Cohen, Kamarck, & Mermelstein, 1983).

- DASS-21: scale designed to assess negative mood using three subscales: stress, anxiety and depression. The scale has 21 items scored on a Likert-type scale from 0 to 3. Reliability studies show very good internal consistency with Cronbach's alpha values of 0.93 (Henry, & Crawford, 2005).

➢ **PAIN EVALUATION:**

- Chronic Pain Grade Scale (CPGS; 90): is a multidimensional measure designed to assess two dimensions of chronic pain severity: pain intensity and pain-induced disability. It's a widely used measure for the evaluation of chronic musculoskeletal pain and back pain. The scale is made up of 7 items scored on a Likert scale from 0 to 10. Reliability studies have shown Crombach's alpha values of 0.74 (Von Korff, Ormel, Keefe & Dworkin, 1992).

- Brief Pain Inventory (BPI): measure designed to evaluate both the intensity of pain (sensory dimension) and the interference of pain in the patient's life (reactive dimension) in patients with chronic pain caused by osteoarthritis, back pain and cancer. It also assesses pain location, pain medication, and pain relief in the past 24 hours. Reliability studies show Cronbach's alpha values ranging between 0.77 and 0.91 (Jensen, Thornby, & Shanti, 2004).

- Chronic Pain Acceptance Questionnaire-Revised (CPAQ-R): instrument designed to evaluate pain acceptance.

Pain acceptance is a construct opposed to attempts to avoid or try to control pain that generate hyperfocusing and maximise feelings of pain. This scale is broken down into two subscales: commitment to activities (pursuit of activities that you feel are important in your life despite pain) and pain disposition (recognition that avoidance and attempts to control are often unsuccessful and increase pain). The scale is made up of 20 items scored on a Likert-type scale from 0 to 6. Reliability studies show good internal consistency with Cronbach's alpha values of 0.82 for the Commitment to Activities scale and 0.78 for the Pain Predisposition scale (McCraken, Vowles, & Eccleston, 2004).

- Pain Catastrophising Scale (PCS): assesses the tendency to magnify the threat of a painful stimulus and the feeling of helplessness in the presence of pain, as well as the inability to prevent or inhibit thoughts related to pain (both before, during, and after the painful event). This instrument is divided into three subscales: pain rumination, pain magnification and feeling of hopelessness when managing pain. The scale is made up of 13 items scored on a Likert-type scale from 0 to 4. Reliability studies show good internal consistency with Cronbach's alpha values ranging between 0.87 and 0.93 (Sullivan Bishop, & Pivik, 1995).

- Brief Fatigue Inventory (BFI): the objective of this scale is to evaluate the severity of fatigue and the impact of pain-induced fatigue on the way people operate on a day-to-day basis. The scale is made up of 9 items scored on a Likert-type scale from 0 to 10. Reliability studies show very good internal consistency with Cronbach's alpha values ranging between 0.82 and 0.97 (Mendoza et al., 1999).

- Pain Self–Efficacy Questionnaire (PSEQ): assesses the extent to which a person in pain believes they are able to carry out their daily activities regardless of their pain. The scale is made up of 10 items scored on a Likert-type scale from 0 to 6. Reliability studies show very good internal consistency with Cronbach's alpha values of 0.92 (Nicholas, 1989; Nicholas, 2007).

- Survey of Pain Attitudes - Brief (SOPA-B): assesses the patient's attitude to seven dimensions of the experience of chronic pain: pain control, disability associated with pain, key medical features of pain, requesting the help of others when dealing with pain,

pain medication, the influence of emotions on pain and pain as an indicator of physical harm. The scale is made up of 30 items scored on a Likert-type scale from 0 to 4. Reliability studies show good internal consistency with values ranging between 0.70 and 0.83 for the different subscales (Jensen et al., 1987; Tait, & Chibnall, 1997).

- Pain Coping Inventory (PC): instrument designed to evaluate the pain coping strategies used by patients with chronic pain. The inventory includes six subscales that assess the following components: pain transformation, distraction, reduction in requests, withdrawal, worry and rest. In turn, these components can be grouped into two dimensions: active and passive coping with pain. The scale is made up of 34 items scored on a Likert-type scale from 1 to 4. Reliability studies show good internal consistency with values greater than 0.70 for the different subscales (Kraaimaat, & Evers, 2003).

➢ **FUNCTIONALITY / DISABILITY:**

- Short Form-36 Bodily Pain Scale (SF-36 BPS): is one of the eight subscales in questionnaire SF-36 (Ware, Kosinski & Keller, 1994) designed as a generic health status assessment for population studies. Subscale SF-36 BPS is made up of two items that measure the interference of pain in daily activities (McHorney, Ware & Raczek, 1993). Reliability studies show internal Cronbach's alpha consistency values that range between 0.72 and 0.77 (Quintana et al., 2005; Escobar et al., 2007).

- Pain and Sleep Questionnaire (PSQ): evaluates the impact of chronic pain on quality of sleep. The scale is made up of 8 items scored on a visual analogue scale from 0 to 100. Reliability studies show adequate internal consistency (Peloso et al., 2000; Watson, 1998).

➢ **WELL BEING:**

- Satisfaction with Life questionnaire (SWLS): a short 5-item scale designed to measure a person's overall cognitive judgments with regard to their life satisfaction. Reliability studies show sound internal consistency with Cronbach's alpha values of 0.78 (Diener, Emmons, Larsen, & Griffin, 1985).

- WHO-5 Well Being Index: 5-item scale designed to assess the subjective psychological well being of the person. Reliability studies show adequate internal consistency (Bech, 2004; Topp et al., 2015).

8.3 COVARIATES

1. Assessment of involvement in the psycho-educational programme: daily practice and attendance.

Questionnaire REFERENCES

Von Korff M, Ormel J, Keefe FJ, Dworkin SF. Grading the severity of chronic pain. Pain 1992;50:133–49.

Ware JE, Kosinski M, Keller SK. SF-36 physical and mental health summary scales: a user’s manual. Boston: The Health Institute; 1994.

McHorney CA, Ware JE, Raczek AE. The MOS 36-Item Short-Form Health Survey (SF-36) II: psychometric and clinical tests of validity in measuring physical and mental health constructs. Med Care 1993;31: 247–63.

Quintana JM, Escobar A, Bilbao A, Arostegui I, Lafuente I, Vidaurreta I. Responsiveness and clinically important differences for the WOMAC and SF-36 after hip joint replacement. Osteoarthritis Cartilage 2005;13:1076–83.

Escobar A, Quintana JM, Bilbao A, Arostegui I, Lafuente I, Vidaurreta I. Responsiveness and clinically important differences for the WOMAC and SF-36 after total knee replacement. Osteoarthritis Cartilage 2007;15:273–80.

Tan, G., Jensen, M. P., Thornby, J. I., & Shanti, B. F. (2004). Validation of the Brief Pain Inventory for chronic nonmalignant pain. The Journal of Pain, 5(2), 133-137.)

Cohen, S., Kamarck, T., & Mermelstein, R. (1983). A global measure of perceived stress. *Journal of Health and Social Behavior, 24*, 385-396.

Henry, J. D., & Crawford, J. R. (2005). The short-form version of the Depression Anxiety Stress Scales (DASS-21): Construct validity and normative data in a large non-clinical sample. British journal of clinical psychology, 44(2), 227-239.
