# Supplementary material for: The effect of mindfulness on the inflammatory, psychological and biomechanical domains of adult patients with low back pain: A randomized controlled clinical trial
Source: PLoS One. 2022 Nov 9;17(11):e0276734. doi: 10.1371/journal.pone.0276734 (PMC9645607; doi:10.1371/journal.pone.0276734)
Supplement: S5 File — (PDF) [file pone.0276734.s005.pdf]

8.4 Evaluation of the response

The database of each participant consists of 3 blocks of measures: demographic, psychological, and biological.

1.- Pre-processing: The missing values, i.e. those that have not been provided by the participant or, in the case of biological measures, that could not be measured, will be estimated by means of a non-linear multivariate data imputation algorithm. Only the values of the "post" means, those taken after having carried out the program, will be imputed.

2.- Dependence relationships between variables. The linear correlation between each pair of variables is estimated for the group in the conditions Pre, before carrying out the MBI or control program, and Post, after the conclusion of these programs. The correlation coefficient is estimated using the Kendall algorithm, which is less sensitive to statistical outliers than the Pearson correlation coefficient. The correlation will be taken as that which meets the statistical criteria of 0.01 over the p-value. In this way we have a semi-binary matrix where the element (i,j) of the matrix will be 0 in case of non-significant correlation or the corresponding correlation value between the variables of positions i and j. The matrix will be symmetric and with null diagonal.

3.-The relationship matrices between variables will be considered as networks or graphs, thus allowing the estimation of topological parameters such as the determination of which variables have a greater intermediary role, greater grouping coefficient or greater information diffusion capacity in the network, as well as global parameters such as the efficiency of the network. Two networks are considered: psychological network, demographic network and biological network, to later consider a multi-layer network composed of the three previous networks and their relationships. As a topological statistic, the measurements will be normalized to those generated by a random network that meets the criteria of the same number of nodes and connections, but with architectures generated by the thousand-fold shuffling method, in order to establish a threshold of statistical significance.

4.- Using artificial intelligence learning machines, the predictive capacity of this network will be evaluated, with the aim of extracting biomarkers of the psycho-bio-social model of discopathy and testing the effectiveness of stress reduction programs based on mindfulness. In this step, the changes induced by the experimental and control

groups are compared experimental and control groups, for the validation of the variables hypothesized as mediators of well-being.

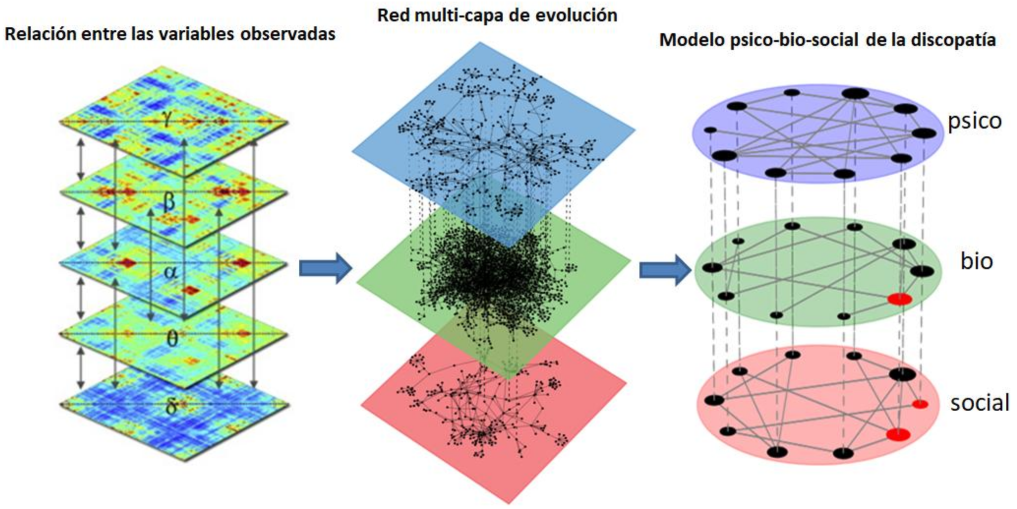

Figure: The relationship between all the variables measured, psychological, demographic-social and biological, provides us with a set of matrices with the correlation coefficients between them (left panel). This information is treated as 3 networks of different nature that connect between them (central panel) to subsequently (right panel) extract which variables have been determinant in the evolution of the participants in the mindfulness-based stress reduction program.
